# Benchmarking batch-effect correction in clinical bead-based multiplex immunoassays: a framework and web tool for cytokine and soluble immune checkpoint data

**DOI:** 10.3389/fimmu.2026.1944271

**Published:** 2026-10-08

**Authors:** Urko Aguirre-Larracoechea, Laura Hurtado-Navarro, Julia del Prado-Montero, Ramón Cantero-Cid, Eloisa Urrechaga-Igartua, Verónica Terrón-Arcos, Jesús Fernández-Felipe, Laura Córdoba, José G. Rigor-Pérez, Rebeca Abad-Moret, Esteban Díaz-Serrano, Roberto Lozano-Rodríguez, Carlos del Fresno, Eduardo López-Collazo

**Affiliations:** 1 Research Unit, Osakidetza Basque Health Service, Barrualde-Galdakao Integrated Health Organization, Galdakao-Usansolo Hospital, Galdakao, Spain; 2 Kronikgune Institute for Health Services Research, Bilbao, Spain; 3 Network for Research on Chronicity, Primary Care, and Health Promotion (RICAPPS), Galdakao, Spain; 4 The Innate Immune Response Group, IdiPAZ, La Paz University Hospital, Madrid, Spain; 5 Tumour Immunology Laboratory, IdiPAZ, La Paz University Hospital, Madrid, Spain; 6 Digestive Surgery Service, La Paz University Hospital, Madrid, Spain; 7 Digestive Surgery Department, Faculty of Medicine, Universidad Autónoma de Madrid, Madrid, Spain; 8 Translational Research and Innovation in Surgery Group, La Paz University Hospital, Madrid, Spain; 9 CORE Laboratory, Hospital Galdakao-Usansolo, Galdakao, Vizcaya, Spain; 10 BioBizkaia Health Research Institute, Barakaldo, Vizcaya, Spain; 11 Faculty of Health Sciences, UNIE University, Madrid, Spain; 12 Immunomodulation Laboratory, IdiPAZ, La Paz University Hospital, Madrid, Spain; 13 CIBER of Respiratory Diseases (CIBERES), Madrid, Spain

**Keywords:** batch-effect, btma_quantnorm, cytokines, immune checkpoints, intraclass correlation coefficient, quantile normalization

## Abstract

**Background:**

Batch effects represent a major source of non-biological variability in clinical bead-based multiplex immunoassays (BBMIs), particularly when samples are processed across different analytical days and when measurements include a substantial proportion of values at or near the limit of detection (LOD).

**Methods:**

Here, we developed and evaluated a comparative framework for reducing inter-batch variability in plasma cytokines with the Essential Cytokines Panel and soluble immune checkpoints (sICs) measurement with the Immune Checkpoint Panel. We also implemented this workflow in a freely accessible R Shiny-based web application that enables interactive batch-effect correction, visualization of correction performance, and export of harmonized datasets. Twenty-five proteins were quantified in 127 clinical samples processed across three analytical days: day 1, *n* = 26; day 2, *n* = 72; and day 3, *n* = 29. Seven batch-correction approaches were evaluated: median-centering, *ComBat*, and five methods from the *batchtma* R package: *btma_simple*, *btma_standardize*, *btma_ipw*, *btma_quantreg* and *btma_quantnorm*. Each method was assessed under two preprocessing strategies: log_2_(x+1) transformation and analysis of raw concentrations expressed in pg/mL values. Correction performance was evaluated using the intraclass correlation coefficient (ICC), Kruskal-Wallis test, and inter-batch coefficient of variation (CV).

**Results:**

Before correction, ICC values were 0.207 for the Essential Cytokines Panel and 0.362 for the Immune Checkpoint Panel; furthermore, 13/13 and 12/12 markers, respectively, showed significant inter-day differences. *btma_quantnorm* achieved the largest and most consistent improvements across both panels and preprocessing strategies, reducing mean ICC values to ≤0.004 corresponding to a ≥98.1% reduction. After correction, only 0–1 markers retained significant Kruskal-Wallis results, and *btma_quantnorm* yielded the lowest inter-day CV for the Immune Checkpoint Panel (80.9%). Importantly, this method does not rely on normality assumptions and showed stable performance across the analytical workflows evaluated.

**Conclusion:**

These findings support *btma_quantnorm* as a practical batch-correction approach for clinical BBMI dataset affected by inter-batch variability and LOD-related distributional constraints. The accompanying web-based tool translates this benchmarking framework into an accessible resource for applying, comparing and evaluating batch-correction in multiplex immunoassay datasets.

## 1 Introduction

Colorectal cancer (CRC) remains a major global health challenge, and its clinical management increasingly depends on the identification of robust circulating biomarkers capable of capturing tumor heterogeneity and supporting patient stratification (1, 2). Liquid biopsies approaches offer a minimally invasive alternative for screening, disease monitoring and assessment of treatment response. However, their clinical utility depends critically on the analytical reliability, traceability and reproducibility of the assays used (3, 4). In this context, circulating immune-related proteins, including cytokines, chemokines, and soluble immune checkpoints (sICs), have emerged as informative indicators of the tumor-host interface (5, 6). These mediators can provide dynamic information on immune activation, tumor progression and therapeutic response. Nevertheless, their low physiological concentrations and the complexity of the blood matrix require highly sensitive analytical platforms with rigorous control of technical variability (7–9).

Single-analyte enzyme-linked immunosorbent assays (ELISAs) have traditionally been used for protein quantification, but their throughput limitations and relatively high sample volume requirements constrain their applicability in large-scale clinical cohorts (10–12). Bead-based multiplexed immunoassays (BBMI) analyzed by flow cytometry, such as the LEGENDplex™ platform, partly overcome these limitations by enabling the simultaneous measurement of multiple analytes in small sample volumes. However, the increased analytical complexity of multiplex platforms introduces specific challenges that must be addressed before these assays can be reliably incorporated into translational and clinical laboratory workflows.

One of the most relevant limitations of BBMI data is their susceptibility to batch-effects, defined as systematic non-biological variation introduced by factors such as the day of analysis, operator, reagent lot, sample handling, or instrument performance (13). In multi-day clinical studies, uncorrected batch effects may introduce substantial analytical bias, inducing spurious clustering in multivariate analyses, and leading to erroneous biological conclusions (14, 15). This issue is particularly important in BBMI datasets for two reasons. First, the bead-based format is more vulnerable to operator- and instrument-dependent variability than conventional single-analyte assays, as the fluorescence detection system is sensitive to daily instrument calibration and environmental fluctuations (16). Second, immune mediator measurements frequently contain a high proportion of values at or below the lower limit of detection (LOD), resulting in zero-inflated or bimodal distributions that challenge the normality assumptions of parametric correction methods (17).

Several statistical strategies have been proposed to mitigate inter-assay or inter-batch variability. Model-based approaches, including mixed-effects modelling, can account for batch-structure directly within the inferential framework and are theoretically well justified (13). However, they require the batch variable to be consistently incorporated throughout all downstream analyses, which may complicate their implementation in multi-stage clinical workflows. Alternatively, post-analytical correction methods can be applied before downstream analyses to generate harmonized datasets. Among these, ComBat, originally developed for microarray gene expression data within an empirical Bayes framework (18), remains one of the most widely used batch-correction approaches in molecular profiling and is increasingly applied to protein quantification datasets (19). Nevertheless, its reliance on Gaussian distributional assumptions may limit its reliability for immunoassay panels with extensive LOD censoring. Alternative statistical frameworks, including location adjustments, *z*-score standardization, inverse probability weighting, quantile regression, and distribution-matching by quantile normalization, may offer superior performance in this specific analytical context (20).

Despite the increasing use of BBMI platforms in translational and clinical research, systematic head-to-head evaluations of batch-correction strategies for multiple proteins’ panels remain limited, particularly for sICs and cytokine datasets (21). This lack of comparative evidence represents an important barrier to the standardization of BBMI-based biomarker studies and to the comparability of results across analytical batches, laboratories and clinical cohorts (22, 23). To address this gap, we evaluated seven batch-correction methods under two preprocessing strategies in 127 clinical samples, in which 25 circulating immune-related proteins were quantified across three analytical batches. To facilitate the implementation and comparison of these approaches, we have also developed a freely accessible R Shiny-based web application. This allows users to apply all the correction methods and preprocessing strategies evaluated in this study, visualize their performance and export harmonized datasets. Our aim was to identify a practical analytical framework for reducing inter-batch variability in BBMI data affected by LOD censoring and to support more reliable protein quantification in clinical immunology and laboratory medicine.

## 2 Materials and methods

### 2.1 Participant recruitment and eligibility criteria

Peripheral blood samples were obtained from colorectal cancer (CRC) patients (n = 127) at the Colorectal Surgery Department of La Paz University Hospital (Madrid, Spain), following the acquisition of written informed consent and in accordance with institutional ethical guidelines (HULP-PI-1958).

All participants had a confirmed CRC diagnosis prior to enrolment and were evaluated in the outpatient clinic before inclusion. Each case was subsequently reviewed by the hospital’s Colorectal Tumor Committee.

Blood samples were collected upon hospital admission, approximately 24 hours before surgery and prior to the initiation of any treatment. Detailed clinical information for the study cohort is provided in **Supplementary Table 1**. To assess potential clinical imbalances across analytical batches, the demographic and clinicopathological variables were compared within and between batches.

### 2.2 Plasma collection

Plasma was obtained from EDTA-anticoagulated venous blood using either density gradient centrifugation with a Ficoll-Plus solution (GE Healthcare Bio-Sciences, Uppsala, Sweden), or direct centrifugation at 3.000 rpm for 15 minutes. The plasma fraction was carefully collected, aliquoted and stored at −80 °C until analysis. The aliquots used for the present analysis were thawed once at 4 °C immediately before measurement and clarified by centrifugation at 15.000xg for 10 minutes to remove any remaining cellular debris. Available sample collection dates were reviewed to estimate storage duration before analysis.

### 2.3 Soluble immune checkpoints and cytokine detection assay

Plasma concentrations of soluble immune checkpoints (sICs), cytokines and chemokines were quantified using LEGENDplex™ bead-based immunoassays (BioLegend, San Diego, CA, USA), following the manufacturer’s instructions.

Briefly, the assay is based on a multiplex bead-based sandwich immunoassay. In this assay, distinct bead populations are coated with analyte-specific capture antibodies. These bead populations are differentiated by size and internal fluorescence intensities. Following incubation with plasma samples, target analytes bind to their corresponding capture beads and are subsequently detected using biotinylated detection antibodies and streptavidin-phycoerythrin (SA-PE), generating a fluorescent signal proportional to analyte concentration. The resolution of individual bead populations is facilitated by the implementation of flow cytometry, a technique that enables the concurrent quantification of multiple analytes within a single sample.

The measurement of sICs was conducted using the Human Immune Checkpoint Panel 1, referred through the manuscript as the ‘Immune Checkpoint Panel’ (catalogue number 740867), which includes sCD25, s4-1BB, sCD27, sCD86, sCTLA-4, sPD-L1, sPD-L2, sPD-1, sTIM-3, sLAG-3, Galectin-9, and sTGF-β. The quantification of cytokines was performed using the Human Essential Cytokines Panel, referred through the manuscript as ‘Essential Cytokines Panel’, which includes interleukin (IL)-1β, IL-2, IL-4, IL-6, IL-8, IL-10, IL-12p70, IL-17A, IFN-γ, TNF-α, CCL2, CXCL10 and free active TGF-β.

The acquisition of data was conducted using a FACSCalibur flow cytometer (BD Biosciences, Franklin Lakes, NJ, USA), and the subsequent analysis was performed with the assistance of LEGENDplex™ software (v.8, BioLegend).

### 2.4 Study samples and batch structure

A total of 127 clinical CRC samples were analyzed across three experimental runs: batch 1 (n =26, 20.5%), batch 2 (n =72, 56.7%), and batch 3 (n =29, 22.8%). This distribution reflects the unequal batch sizes commonly encountered in clinical cohort studies, in which sample availability, reagent lots, and acquisition days may introduce batch-related variability.

Prior to batch-correction, a censoring assessment was performed to identify markers with ≥15% of measurements at the LOD and/or upper limit of quantification (ULOQ), following established recommendations (25). Twelve out of 25 markers exceeded this threshold, with censoring rates ranging from 18.9% for IL-10 to 57.5% for sPD-L2 at ULOQ (**Supplementary Table 2**).

For these censored observations, no secondary statistical imputation was applied to measurements outside the quantifiable range before transformation or batch correction. Instead, concentration values were analyzed as exported by LEGENDplex™ software v.8. Values reported numerically at the lower analytical boundary were retained at their exported LOD-associated value, whilst measurements returned as zero were retained as zero. Measurements exceeding the upper quantification range and reported at the assay ceiling were retained at the corresponding ULOQ; this applied notably to sPD-L2, for which the upper boundary was 5,000 pg/mL. Consequently, repeated boundary values should be interpreted as censored observations rather than fully independent quantitative measurements. The proportion of affected samples, repeated boundary value, number of zeros and censoring type for each affected analyte are reported in **Supplementary Table 2**.

### 2.5 Batch-correction methods

Seven methods were compared (**Table 1**): median centering; *btma_simple* (mean-centering); *btma_standardize* (within-batch z-score); *ComBat* [empirical Bayes modelling of additive and multiplicative batch-effects (24)]; *btma_ipw* (inverse probability weighting by batch propensity); *btma_quantreg* (quantile regression); and *btma_quantnorm* (quantile normalization, forcing identical empirical distributions across batches). The five btma methods belong to the batchtma R package (0.2.0 version); *ComBat* was applied via the sva Bioconductor package (3.23 release). Additional details on the rationale and implementation of each method are provided in **Supplementary Table 3**. All analyses were conducted in R version 4.3.

**Table 1:** Batch-correction methods evaluated in this study.

Family	Method	Principle	Normality assumption	log_2_ in strategy A
Location	Median	Subtracts the batch median from each observation; robust to LOD accumulation	No	No
Location	*btma_simple*	Mean-centering with restoration of the global mean	Implicit	Yes
Standardization	*btma_standardize*	Within-batch z-score standardization (mean and variance)	Implicit	Yes
Empirical Bayes	*ComBat*	Additive and multiplicative batch effects via empirical Bayes shrinkage (1).	Yes	Yes
Weighting	*btma_ipw*	Inverse probability weighting by batch propensity score	No	Yes
Quantile regression	*btma_quantreg*	Quantile regression capturing batch-effects at each distributional quantile	No	Yes
Distribution	*btma_quantnorm*	Forces identical empirical distribution across batches; no distributional assumption	No	No

Methods marked ‘Yes’ in the log_2_ column are applied after log_2_(x+1) transformation in strategy A and back-transformed thereafter.

Two preprocessing strategies were considered. In Strategy A, a log_2_(x+1) transformation was applied before correction for the five parametric methods that may be affected by scale and distributional properties*: btma_simple, btma_standardize, btma_ipw, btma_quantreg* and *ComBat*. Corrected values were subsequently back-transformed using back-transformation (2^x − 1). In Strategy B, all methods were applied directly to raw concentrations values expressed in pg/mL. Median-centering and *btma_quantnorm* were applied without transformation in both strategies. When correction generated residual negative values in Strategy B, these values were truncated to zero.

### 2.6 Performance metrics

Batch-effect was quantified by the batch-associated intraclass correlation coefficient (ICC), defined as ICC=σ²b/(σ²b+σ²e) and estimated from a one-way random-effects model with analytical batch as the grouping factor, thereby estimating the proportion of total variance in marker concentration attributable to batch membership. Lower post-correction ICC values indicate lower residual batch-related variance. Because estimates were derived from only three analytical batches, ICC values are interpreted as descriptive measures of batch-associated variance rather than precise estimates of a population-level parameter, and are reported continuously, alongside their 95% confidence intervals obtained by stratified bootstrap with 500 iterations, rather than dichotomized against a fixed threshold.

Residual inter-day differences were assessed for each marker using the Kruskal-Wallis test, given the non-parametric distribution of the data. To account for the simultaneous testing of analytes within each panel, *p*-values were adjusted using the Benjamini–Hochberg false-discovery rate (FDR) procedure separately for each combination of panel, correction method, and preprocessing strategy. The number of markers with an FDR-adjusted *q*-value <0.05 was used as a secondary performance metric. Nominal *p*-values are provided for completeness but were not used as the principal criterion for declaring residual batch-associated differences. The inter-day coefficient of variation, calculated as CV%=SD/mean×100 and averaged across markers, provided a complementary measure of batch-associated variability. Because CV is strongly influenced by the mean and can become unstable when corrected concentrations approach zero, particularly in heavily censored markers, it was interpreted cautiously and not used as a standalone criterion for method selection. In **Table 2**, CV values are therefore reported as median [Q1–Q3] across markers to reduce sensitivity to extreme values. Principal component analysis (PCA) was performed on auto scaled data to visualize batch-structure before and after correction.

**Table 2:** Comparative performance of all seven batch-correction methods under strategy A (log_2_ transformation) and strategy B (raw pg/mL), for the essential cytokines panel and the immune checkpoint panel.

Method	ICC-A	KW-A, n [q<0.05] (nominal)	CV-A, % median [Q1–Q3]	ICC-B	KW-B, n [q<0.05] (nominal)	CV-B, % median [Q1–Q3]	ΔICC (B−A)	Best
Essential cytokines panel								
btma_quantnorm	0.004	1 [4]	153.7 [109.0–214.8]	0.004	1 [4]	153.7 [109.0–214.8]	0.000	=
btma_quantreg	0.005	7 [7]	338.1 [158.6–373.1]	0.032	7 [7]	552.1 [370.1–740.8]	0.027	A
ComBat	0.040	6 [7]	196.5 [123.5–267.9]	0.042	11 [11]	141.6 [105.5–241.7]	0.002	A
Median	0.041	1 [3]	437.9 [357.2–530.2]	0.041	1 [3]	437.9 [357.2–530.2]	0.000	=
btma_simple	0.051	8 [8]	190.0 [175.9–268.8]	0.006	11 [11]	178.8 [140.7–218.6]	−0.045	B
btma_standardize	0.051	8 [8]	190.0 [175.9–268.8]	0.006	11 [11]	178.8 [140.7–218.6]	−0.045	B
btma_ipw	0.051	8 [8]	190.0 [175.9–268.8]	0.006	11 [11]	178.8 [140.7–218.6]	−0.045	B
Original	0.207	13 [13]	166.8 [123.6–292.3]	0.207	13 [13]	166.8 [123.6–292.3]	0.000	—
Immune checkpoints panel								
btma_quantnorm	0.004	0 [0]	66.9 [52.3–80.9]	0.004	0 [0]	66.9 [52.3–80.9]	0.000	=
btma_quantreg	0.001	1 [1]	108.1 [93.4–121.8]	0.004	2 [2]	93.7 [81.7–123.8]	0.003	A
ComBat	0.013	2 [2]	73.2 [57.5–89.8]	0.002	2 [2]	76.6 [55.0–82.7]	−0.011	B
btma_simple	0.041	2 [2]	75.7 [69.7–95.7]	0.005	5 [6]	92.0 [65.8–102.9]	−0.036	B
btma_standardize	0.041	2 [2]	75.7 [69.7–95.7]	0.005	5 [6]	92.0 [65.8–102.9]	−0.036	B
btma_ipw	0.041	2 [2]	75.7 [69.7–95.7]	0.005	5 [6]	92.0 [65.8–102.9]	−0.036	B
Median	0.043	0 [0]	384.4 [354.2–451.9]	0.043	0 [0]	384.4 [354.2–451.9]	0.000	=
Original	0.362	12 [12]	89.7 [72.0–107.6]	0.362	12 [12]	89.7 [72.0–107.6]	0.000	—

ICC, intraclass correlation coefficient (batch-associated); KW, number of markers with Kruskal-Wallis test significant at q<0.05 after Benjamini-Hochberg FDR correction, with the nominal p<0.05 count shown in brackets for completeness; CV, inter-day coefficient of variation (%), reported as median [Q1-Q3] across markers to reduce sensitivity to extreme denominators; ΔICC (B-A), difference in ICC between Strategy B (raw concentration) and Strategy A (log2-transformed); Best, strategy (A or B) associated with the lower ICC for that method (“=“ indicates identical performance across strategies; “—” indicates the uncorrected Original data, shown for reference).

To further evaluate correction performance beyond the reduction of batch-associated differences, we assessed the preservation of biological signal across all batch-correction methods implemented in the analysis workflow: median normalization, *ComBat, btma_simple, btma_standardize, btma_ipw, btma_quantreg and btma_quantnorm*. Three complementary metrics were computed for each method, using the corrected values generated under the preprocessing strategy described above.

First, preservation of marker–clinical variable associations was assessed by computing Spearman correlation coefficients between each marker and each of 11 clinical variables, both before and after correction, and summarizing agreement between the two sets of coefficients as their Pearson correlation, referred to as “vector correlation”, the median absolute difference between paired coefficients, and the percentage of marker–clinical variable pairs retaining the same direction of association.

Second, preservation of within-batch sample ranking was assessed by computing the Spearman correlation between original and corrected marker values separately within each of the three analytical batches, then averaging across batches and markers.

Third, preservation of the marker–marker correlation structure was assessed by computing the full Spearman correlation matrix among all markers before and after correction and comparing the two matrices via the Pearson correlation of their upper-triangle entries, referred to as the “matrix correlation coefficient”, and the mean absolute change in pairwise correlation coefficients, computed for the full 25-marker panel and separately for each of the Essential Cytokines and Immune Checkpoint panels.

### 2.7 Simulation-based sensitivity analysis

To assess the robustness of the correction-method comparison beyond the specific censoring level, batch structure and sample size of the present cohort, we performed a calibrated simulation study. Marker-specific means, standard deviations and the inter-marker Spearman correlation matrix (log_2_(x+1) scale) were estimated separately for each panel from the real dataset. These parameters were used to generate correlated synthetic marker values via Cholesky decomposition. An additive per-batch, per-marker effect was then applied, followed by target left/right censoring, to approximate the pre-correction batch-associated ICC observed in the real data (0.207 and 0.362 for the Essential Cytokines and Immune Checkpoint panels, respectively). Using this framework, we generated scenarios varying the censoring proportion, the number and balance of analytical batches, and the total sample size, and re-applied all seven correction methods to each scenario. In two additional scenarios, a synthetic covariate with a known true effect on a subset of markers was introduced either as strongly confounded with batch assignment or as independent of batch. These scenarios were used to estimate the extent to which covariate-blind correction could attenuate a true biological signal under adverse confounding conditions. This simulation-based analysis was intended as a complementary robustness assessment of the comparative behavior of the correction methods under controlled assumptions. It does not replace independent technical validation using shared reference material or replicated clinical samples across analytical batches. The simulated scenarios and corresponding results are summarized in the **Supplementary Material**.

### 2.8 Interactive web-based tool for batch-effect correction

To translate the methodological framework developed in this study into a practical and reusable resource for the research community, an interactive web application called ‘BatchCorrector’ was created using the R Shiny framework. It is available at https://uappstats.shinyapps.io/BatchCorrection. This application enables users to upload their own datasets, apply the batch-correction methods and preprocessing strategies evaluated in this study, visualize the performance of the corrections, and export harmonized datasets for subsequent analyses. Although the ‘BatchCorrector’ application is freely accessible to the scientific community, the software underlying the tool is protected under registered intellectual property rights (registration number: 2026RTE00927504).

## 3 Results

### 3.1 Initial assessment of batch-effects in uncorrected data

Before correction, substantial batch-associated differences were observed across both panels (**Figure 1**). In both the Essential Cytokines Panel and the Immune Checkpoint Panel, marker concentration distributions differed markedly between the three analytical batches, indicating systematic inter-batch variability in the original data (**Figures 1A, B**). The marker-level distributions underlying these batch-specific concentration profiles are summarized in **Supplementary Table 4**, expressed as median values with interquartile ranges (P25–P75).

**Figure 1:**
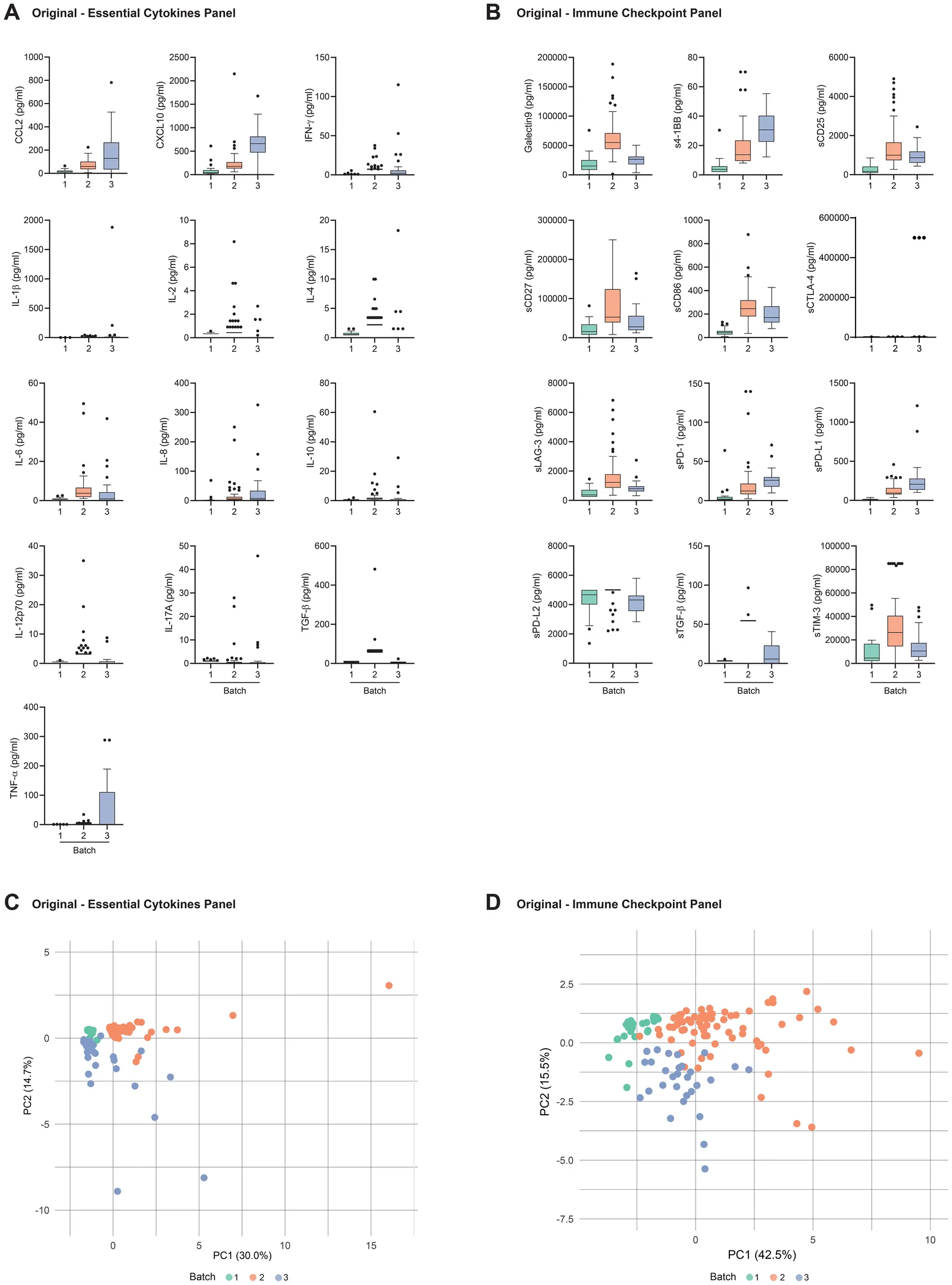
Original data distributions and principal component analysis (PCA) before batch correction. Box plots showing the concentration distributions of each marker across the three analytical batches before correction for the Essential Cytokines **(A)** and the Immune Checkpoint **(B)**. Concentrations are expressed in pg/mL. Principal component analysis (PCA) score plots of the uncorrected data are shown for the Essential Cytokines **(C)** and the Immune Checkpoint **(D)**, with samples colored according to analytical batch.

In the Essential Cytokines Panel, CXCL10, CCL2, and IL-8 exhibited progressively higher concentrations from batch 1 to batch 3, whereas IL-2, IL-4, IL-12p70 and IL-17A reached their highest values in batch 2 and were markedly lower in batch 3. In the Immune Checkpoint Panel, s4-1BB, sPD-1 and sPD-L1 displayed the highest concentrations in batch 3, while sCTLA-4 and sTGF-β were predominantly increased in batch 2. Additional markers, including Galectin-9, sCD25, sCD27 and sTIM-3 also showed noticeable variation across batches.

The mean ICC across markers was 0.207 for the Essential Cytokines Panel, in which all 13 analytes exhibited significant Kruskal-Wallis differences across batches, with an average CV of 231.6%. In the Immune Checkpoint Panel, the mean ICC was 0.362, with significant inter-batch differences in 12 of 12 markers and an average CV of 122.7%. At the marker level, ICC values were highly heterogeneous, ranging from 0.000 for IL-17A (29.9% of values at the LOD) to 0.587 for CXCL10 in the Essential Cytokines Panel. In the Immune Checkpoint Panel, ICC values ranged from 0.149 for sPD-L2 to 0.920 for sTGF-β.

PCA score plots confirm clear batch-dependent clustering in both panels, particularly in the Immune Checkpoint Panel, supporting the presence of marked analytical structure in the uncorrected data (**Figures 1C, D**). This batch-related variability was particularly pronounced in markers with high censoring rates. Notably, 12 markers exceeded the predefined ≥15% censoring threshold, with the highest rates observed for free active TGF-β (55.1%), sCTLA-4 (53.5%), TNF-α (52.8%), and sPD-L2, the latter exhibiting 57.5% of its values at the ULOQ.

Importantly, there were no statistically significant differences in demographic and clinicopathological characteristics across analytical batches, including age, biological sex, tumor site, TNM stage, ASA score, comorbidities, perineural or lymphovascular invasion, and metastatic status (**Supplementary Table 1**). Therefore, the observed variability associated with the different batches was unlikely to be primarily explained by major imbalances in the baseline clinical characteristics of the patients.

Taken together, the combination of significant inter-day differences, heterogeneous ICC values, PCA-based clustering, and high CV values highlights the need for robust post-analytical harmonization before downstream biological or clinical interpretation.

### 3.2 Performance of correction methods

To address these batch-related effects, we evaluated seven correction methods across two preprocessing strategies, with the results summarized in **Table 2**. For each strategy, correction performance was assessed using per-marker ICC plots, Kruskal-Wallis heatmaps, and PCA-based batch-structure (**Figures 2**, **3**).

**Figure 2:**
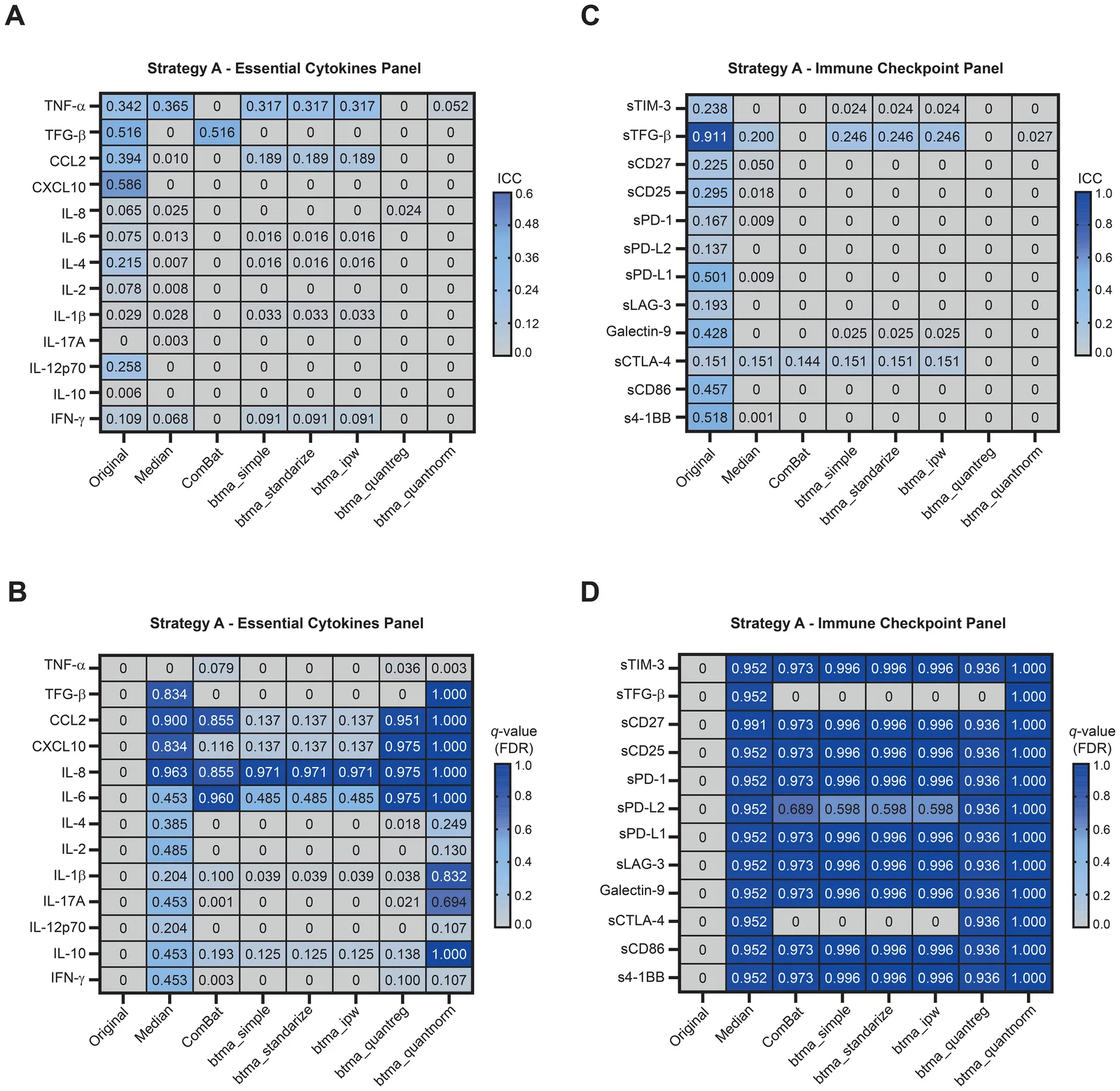
Batch correction performance under strategy **(A)** Per-marker ICC values and Kruskal-Wallis *q*-value heatmaps after correction under Strategy A, based on log_2_(x+1) transformation and back-transformation for selected methods. Panels show the Essential Cytokines **(A, B)** and the Immune Checkpoint **(C, D)**. Lower *q*-values indicate persistent batch-associated differences.

**Figure 3:**
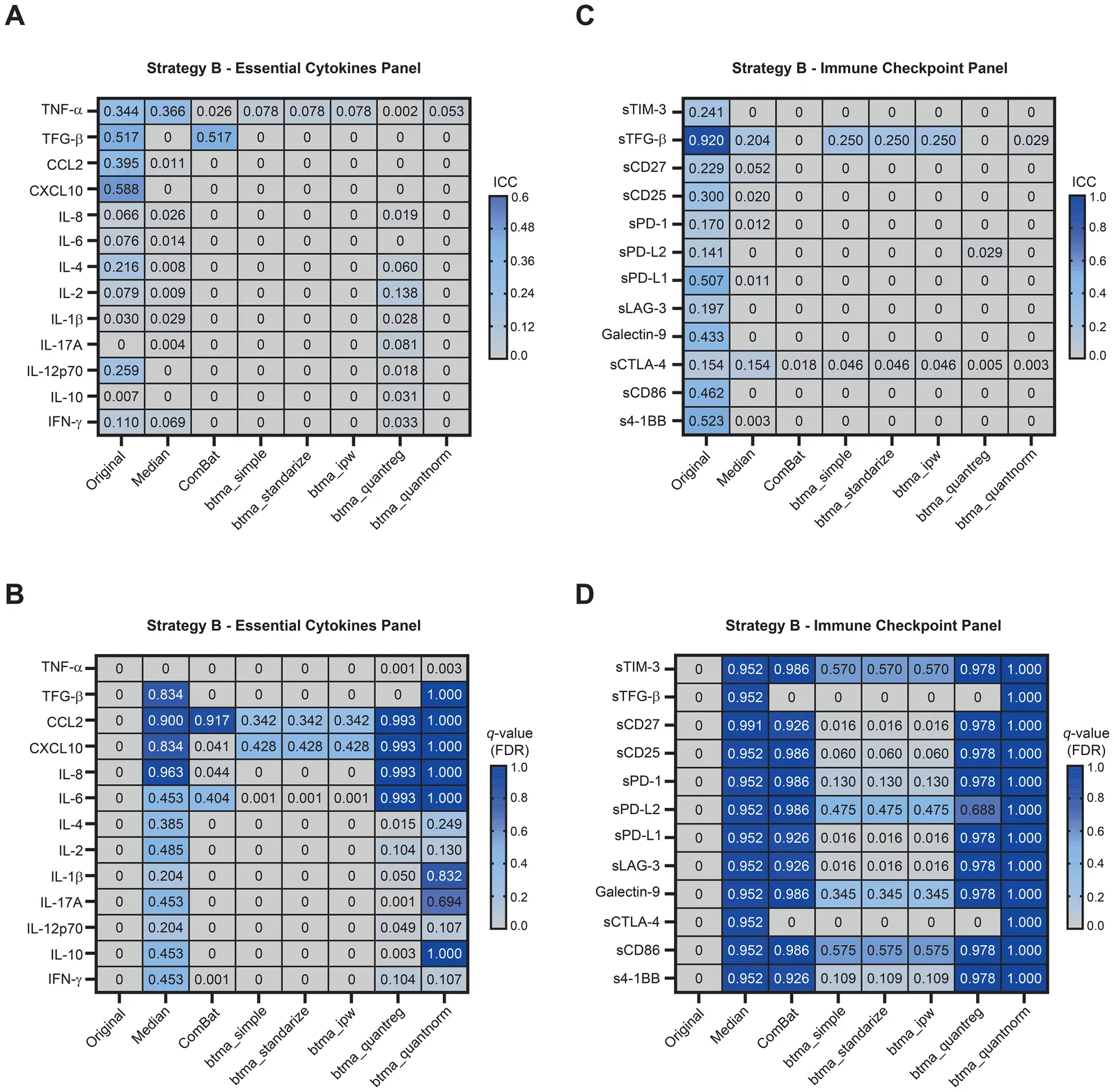
Batch correction performance under Strategy **(B)** Per-marker ICC values and Kruskal-Wallis *q*-value heatmaps after correction under Strategy B, in which methods were applied directly to raw concentration values expressed in pg/mL. Panels show the Essential Cytokines **(A, B)** and the Immune Checkpoint **(C, D)**. Lower *q*-values indicate persistent batch-associated differences.

In Strategy A (log_2_ scale), several methods reduced the batch-associated signal, with performance varying by panel (**Figure 2**). In the Essential Cytokines Panel, *btma_quantnorm* achieved the lowest mean ICC (post-correction ICC = 0.004; 98.1% reduction; one KW-significant marker (FDR *q<*0.05); CV%=172.9%), while *btma_quantreg* showed similar ICC reduction (0.005) but a markedly higher inter-day CV (421.2%), suggesting instability for some low-abundance or near-LOD analytes (**Table 2**, **Figures 2A, B**). Median centering and *ComBat* also reduced batch-effects, though less markedly, with ComBat ranking third (ICC = 0.040; 80.7% reduction; 7 KW-significant markers) (**Table 2**, **Figures 2A, B**).

In the Immune Checkpoint Panel, *btma_quantreg* yielded the lowest mean ICC (0.001; 99.7% reduction), whereas *btma_quantnorm* combined a low ICC (0.004; 98.9% reduction) with higher analytical stability, eliminating all Kruskal–Wallis–significant differences (0 KW-significant markers) and achieving the lowest inter-day CV% (80.9%) (**Table 2**, **Figures 2C, D**). *ComBat* also substantially reduced mean ICC, although some marker-level differences remained (**Figures 2C, D**).

The robustness of these findings was further assessed in Strategy B, in which correction methods were applied directly to raw concentration values expressed in pg/mL (**Figure 3**). *Btma_quantnorm* produced identical results across both strategies, consistent with its transformation-independent distribution-matching strategy (**Figures 2**, **3**). This invariant performance contrasted with the variability observed for *ComBat*. *Combat* showed slightly improved ICC performance for the Immune Checkpoint Panel in the raw-data strategy compared with the log_2_-transformed strategy (0.002 vs 0.013), suggesting that some sICs may not benefit from standard log_2_ transformation (**Figure 2D**, **3D**). The three methods *btma_simple, btma_standardize* and *btma_ipw* produced identical corrected values in both marker panels and both preprocessing strategies (**Figures 2**, **3**).

### 3.3 Method selection

Based on the comparative performance across complementary metrics, *btma_quantnorm* was selected as the harmonization approach for subsequent analyses. This selection was supported by four primary batch-reduction criteria: (i) consistently low mean ICC values across both panels and preprocessing strategies (≤0.004; ≥98% reduction); (ii) absence of residual Kruskal-Wallis-significant inter-batch differences in the Immune Checkpoint Panel, with only one marker retaining significance after FDR correction in the Essential Cytokines Panel; (iii) a favorable inter-day CV%, particularly for the Immune Checkpoint Panel; and (iv) the identical performance regardless of preprocessing strategy. The aggregate ICC comparison further supported this selection, showing that *btma_quantnorm* maintained one of the lowest average ICC values across both strategies while avoiding the instability observed for alternative methods when additional performance metrics were considered (**Figure 4**).

**Figure 4:**
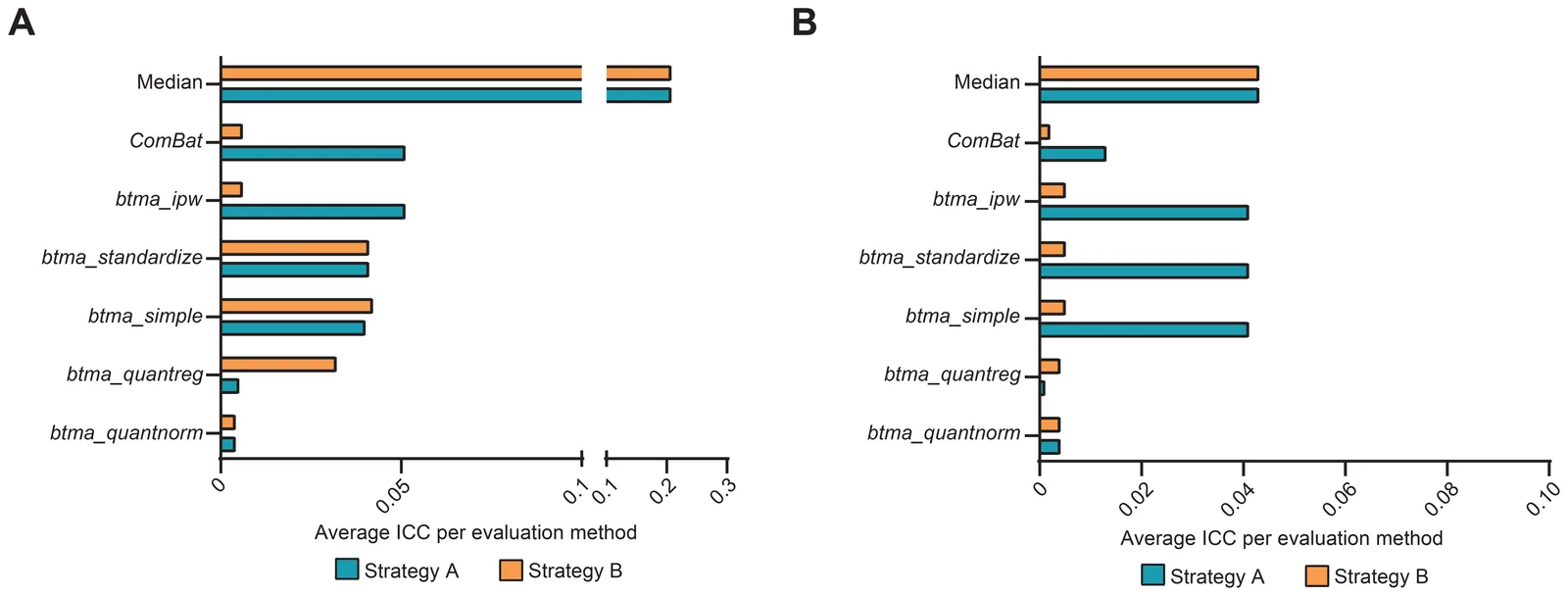
Average ICC after batch correction across preprocessing strategies. Horizontal bar plots showing the average post-correction ICC obtained with each correction method under Strategy A and Strategy B Results are shown separately for the Essential Cytokines **(A)** and the Immune Checkpoint **(B)**. Lower ICC values indicate lower residual batch-related variance after correction.

Following the selection of *btma_quantnorm*, we examined the corrected data structure to assess whether the batch-associated patterns observed in the original dataset had been attenuated following harmonization. **Figure 5A, B** illustrates the post-correction marker distributions across the three analytical batches for the Essential Cytokines Panel and the Immune Checkpoint Panel, respectively. Compared with the uncorrected distributions (**Figures 1A, B**), the corrected profiles exhibited increased overlap between batches for most analytes. This reduction in residual inter-day variability was also supported by the CV heatmaps comparing original and *btma_quantnorm*-corrected data (**Supplementary Figure 1**). Consistently, PCA score plots for both panels showed no evident separation according to analytical batch after *btma_quantnorm* correction, supporting the reduction of batch-associated structure in the harmonized data (**Figures 5C, D**).

**Figure 5:**
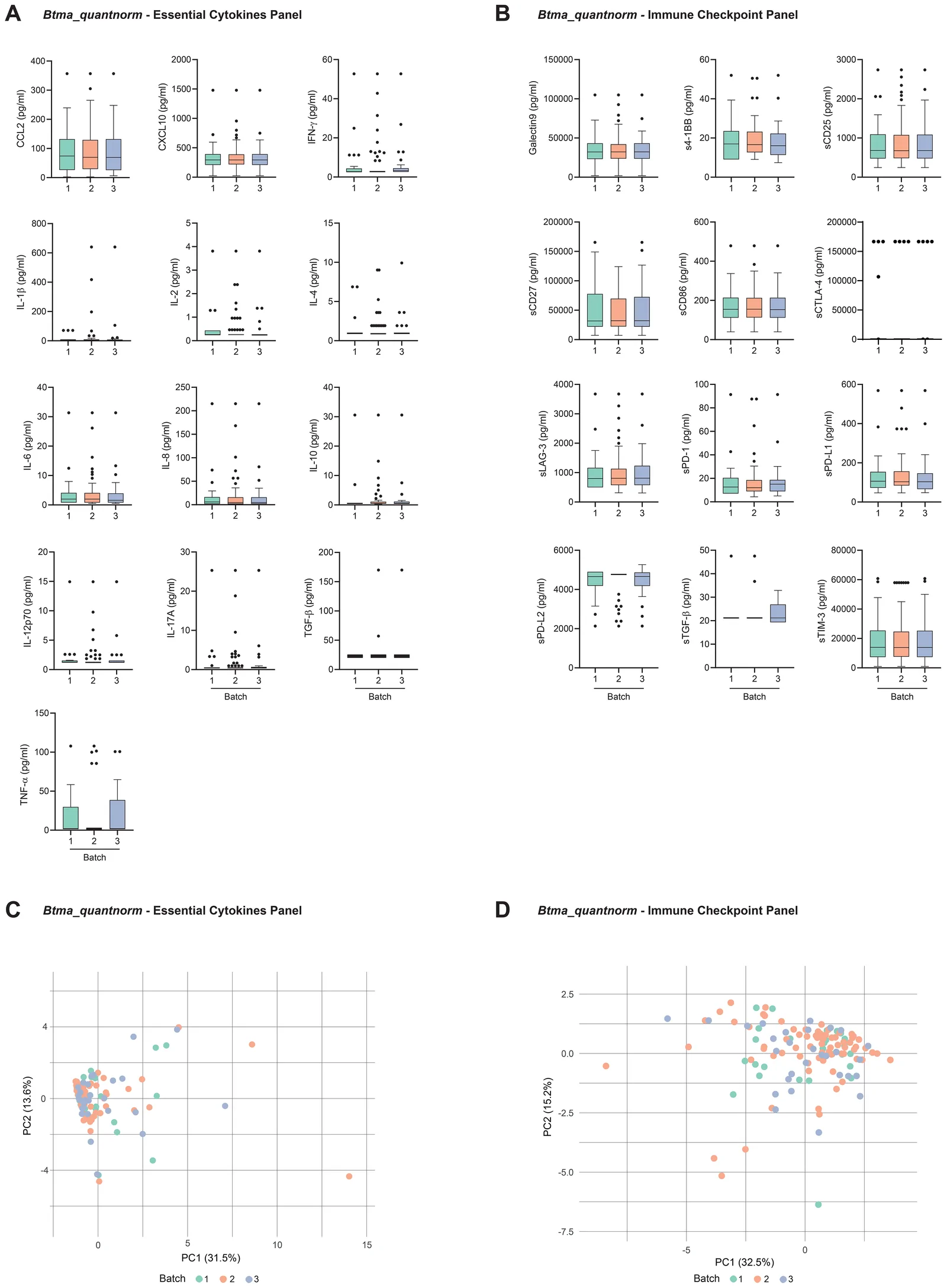
Marker distributions and principal component analysis after *btma_quantnorm* correction. Box plots showing the concentration distributions of each marker across the three analytical batches after *btma_quantnorm* correction for the Essential Cytokines **(A)** and the Immune Checkpoint **(B)**. Concentrations are expressed in pg/mL. Principal component analysis (PCA) score plots of the *btma_quantnorm*-corrected data are shown for the Essential Cytokines **(C)** and the Immune Checkpoint **(D)**, with samples colored according to analytical batch.

To assess the preservation of biological signal across all seven batch-correction methods, we used three complementary criteria: preservation of marker–clinical variable associations, preservation of within-batch sample ranking, and preservation of the marker–marker correlation structure (**Supplementary Tables 5**-**7**). Across the six methods for which all 25 markers could be evaluated, performance was broadly comparable across the three criteria: vector correlation between pre- and post-correction Spearman coefficients ranged from 0.785 to 0.837; mean within-batch rank preservation ranged from 0.998 to 0.9998; and marker correlation-structure preservation ranged from 0.368 to 0.439. No single method outperformed the others across all three dimensions simultaneously. A seventh method, quantile regression-based correction (*btma_quantreg*), showed a nominally higher clinical effect-size preservation score (0.863); however, this method failed to converge for 10 of the 25 markers, particularly those most affected by censoring at the assay’s limit of detection. Therefore, its apparent advantage reflects a smaller, more favorable marker subset (15 of 25) and is not directly comparable to the other six methods.

For the selected *btma_quantnorm* correction, marker–clinical associations were compared before and after harmonization using Spearman correlation analyses. The results of this exploratory assessment were summarized by clinical variable and by nominally significant marker–clinical associations (**Supplementary Tables 8**, **9**). Although certain associations changed their nominal statistical significance status after correction, the analysis did not indicate systematic signal loss or widespread reversal of marker–clinical association patterns after *btma_quantnorm* harmonization.

Taken together, btma_quantnorm performed within the same range as the alternative correction methods evaluated on all three criteria, with no evidence of greater signal loss relative to the alternatives. Given the moderate size of the correlation-structure preservation metric across all methods (0.368–0.439), these findings show comparable performance rather than full preservation of biological signal. To assess whether these findings extend beyond the specific censoring level, batch structure and sample size of the present cohort, the calibrated simulation framework described in the Methods was used to generate eight additional scenarios varying these parameters (**Supplementary Table 10**). Across the scenario–strategy–panel combinations tested, *btma_quantnorm* was the only method with no residual Kruskal-Wallis-significant markers in every combination, including a combined worst-case scenario with five unbalanced batches and ~55% censoring, under which ComBat performance degraded materially (mean ICC increased to 0.040, with a mean of 5.5 markers per combination retaining a significant residual batch-associated difference) (**Supplementary Table 11**).

To estimate, under controlled simulated conditions, how much genuine biological signal could be attenuated by this harmonization strategy, the same simulation framework was used to introduce a synthetic covariate with a known true effect (Cohen’s d ≈ 0.82–0.85 before correction) on a subset of markers, under two conditions: strongly confounded with batch assignment, or distributed independently of it (**Supplementary Table 12**). When the covariate was independent of batch, consistent with the absence of demographic and clinicopathological imbalance observed across batches in the present cohort (**Supplementary Table 1**), every correction method, including *btma_quantnorm*, removed residual batch-associated differences while leaving the true covariate effect materially unchanged (attenuation ≤5%) and statistically significant in every instance tested. Even under the strongest confounding pattern modelled, covariate-blind correction attenuated the true effect by only 16–22%, and statistical significance was retained. These simulation-derived estimates are necessarily specific to the conditions modelled and are presented as a complement to, rather than a substitute for, direct empirical evidence.

### 3.4 The BatchCorrector web application

To facilitate its implementation, this methodological framework was translated into a practical resource for the research community through the development of a web-based interactive application, ‘BatchCorrector’, which is publicly accessible at https://uappstats.shinyapps.io/BatchCorrection/. The application streamlines the complete batch-correction workflow in three main stages (**Figure 6**).

**Figure 6:**
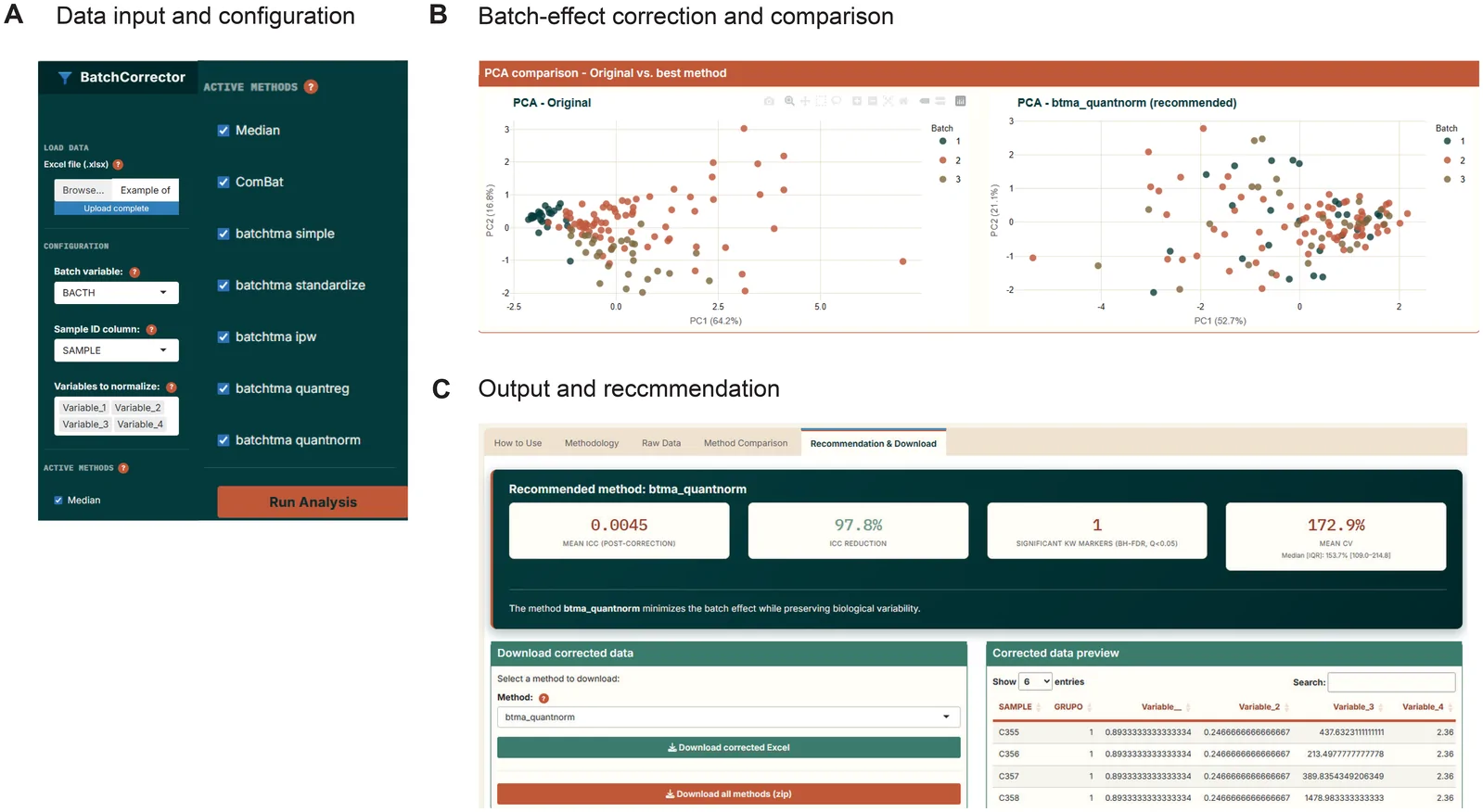
Overview of the ‘BatchCorrector’ web application interface and workflow. **(A)** Data input and configuration panel, where users upload their dataset, define the batch variable and the variables to be corrected, and select one or more of the seven implemented correction methods. **(B)** Batch-effect correction and comparison panel, illustrated by principal component analysis (PCA) plots comparing the original, uncorrected data with the best-performing (recommended) method. **(C)** Output and recommendation panel, summarizing the performance of the recommended method — mean ICC, ICC reduction, number of markers with a significant Kruskal–Wallis test after Benjamini–Hochberg FDR correction (*q* < 0.05), and inter-batch CV (mean and median [IQR]) — and allowing the corrected dataset to be downloaded for the selected method or for all methods at once.

Users first upload their own dataset in Excel or CSV format, define the batch variable, the sample identifier and the variables to be corrected, and select all or a subset of the available correction methods — median-centering, ComBat, btma_simple, btma_standardize, btma_ipw, btma_quantreg and btma_quantnorm (**Figure 6A**). Notably, the underlying correction methods implemented in the tool are not inherently restricted to multiplex immunoassay data and could, in principle, be applied to other multivariate quantitative datasets measured across two or more analytical runs. Application to other analytical platforms or data structures therefore requires independent assessment of the underlying assumptions and performance.

Once the analysis has been run, correction performance can be inspected interactively, for example through principal component analysis (PCA) plots comparing the original data with the recommended method (**Figure 6B**). Additional visual and quantitative outputs, including marker-level boxplots and tables reporting ICC values, Benjamini-Hochberg FDR-adjusted Kruskal-Wallis q-values and inter-batch CV for every evaluated method, are available in the corresponding tabs of the application.

Finally, the recommended method is highlighted together with a summary of its performance — mean ICC, percentage ICC reduction, number of markers retaining a significant Kruskal–Wallis test after Benjamini–Hochberg FDR correction (*q* < 0.05), and inter-batch CV, reported as both the mean and the median [IQR] — and the corrected dataset can be downloaded, for the selected method or for all methods at once, in Excel format, alongside a preview of the corrected data (**Figure 6C**).

Together, these functionalities provide a practical implementation of the comparative framework evaluated in this study, enabling users to apply, compare, and export batch-correction strategies in their own datasets.

## 4 Discussion

This study provides a systematic benchmarking of batch-correction methods for clinical BBMI data, spanning both cytokine and soluble immune checkpoint panels, an analytical context that remains insufficiently explored in the harmonization literature (21–23). The main finding is that *btma_quantnorm* showed the most balanced performance across complementary evaluation metrics and compared favorably with the other approaches evaluated, including the widely used *ComBat* algorithm, in the scenarios evaluated.

The performance of *btma_quantnorm* is consistent with its underlying statistical rationale: quantile normalization reduces distributional differences between batches across the full empirical distribution (20), rather than correcting only location as in mean-centering, or both location and variance, as with z-score standardization and *ComBat* methods. This feature is particularly relevant to BBMI datasets, in which LOD censoring can produce bimodal or zero-inflated distributions that violate Gaussian assumptions and compromise the reliability of correction methods based on parametric models. Nevertheless, distribution-matching approaches such as *btma_quantnorm* carry a potential risk of overcorrection. By mapping batch-specific empirical distributions to a common reference, quantile normalization cannot distinguish technical batch effects from genuine biological differences. Consequently, meaningful biological variation may be attenuated when clinically relevant groups are unevenly distributed across analytical batches. In the present study, this risk was partly mitigated by the absence of statistically significant demographic or clinicopathological differences across batches (**Supplementary Table 1**). Moreover, an exploratory analysis comparing marker–clinical associations before and after *btma_quantnorm* harmonization, based on Spearman correlation coefficients and examined both by clinical variable and by marker, did not indicate systematic signal loss or widespread reversal of association patterns (**Supplementary Tables 8**, **9**). To place this observation in the context of the full set of correction methods evaluated, we further compared the preservation of biological signal across all seven batch-correction methods using three complementary criteria — clinical association preservation, within-batch rank preservation, and marker correlation-structure preservation — and found performance closely clustered across the six fully evaluable methods, with *btma_quantnorm* falling within the same range as the alternative methods across the criteria evaluated (**Supplementary Tables 5**-**7**).

Nonetheless, *btma_quantnorm* should be applied cautiously when batch allocation is known or suspected to be confounded with biological or clinical variables. A calibrated simulation-based sensitivity analysis addressing this scenario directly (**Supplementary Table 10**-**12**) found that, even under a strongly confounded synthetic covariate, *btma_quantnorm* and other covariate-blind methods retained the statistical significance of the true effect in every instance tested, with effect-size attenuation of 16–22%; when the covariate was distributed independently of batch, more consistent with the absence of demographic and clinicopathological imbalance observed here, attenuation was negligible (≤5%). These findings provide reassurance under the simulated conditions but do not eliminate the risk of overcorrection or replace independent validation in datasets with different batch–biology relationships. Future validation studies should therefore explicitly assess the preservation of biological signal, as previously emphasized in investigations examining the effects of batch correction on downstream biological inference (25–27).

The behavior observed for free active TGF-β illustrates the limitation of parametric correction in highly censored BBMI markers. This marker showed a high censoring rate, with 55.1% of samples at the LOD, and retained a substantial post-correction ICC after *ComBat* adjustment (ICC = 0.517). One possible explanation is that empirical Bayes shrinkage may be influenced by the accumulation of identical LOD values, rather than the informative proportion of the distribution. Similar concerns have been raised in proteomic batch-correction benchmarks (19, 28), although the specific context of LOD-heavy BBMI data has been less addressed. This limitation is not exclusive to *ComBat*. More advanced model-based approaches, such as the mixed-effects approach outlined by Clarke et al. (29), can approximately account for batch-structure within the inferential model, but require assumptions about residual distributions and require the batch-variable to be carried forward through downstream analyses. This may be less straightforward when the workflow includes unsupervised steps, such as PCA or clustering, where batch-adjustment cannot always be implemented as a simple covariate. Consequently, a preprocessing strategy that generates a single harmonized dataset and is less dependent on distributional assumptions may be particularly useful for clinical immunoassay workflows.

The 15% LOD censoring threshold applied here was used to identify markers requiring special attention, in line with bioanalytical validation principles and previous observations by Breen et al. (30), on the statistical value of raw fluorescence signals in Luminex xMAP-based multiplex immunoassays. Our data supports the practical relevance of this threshold: the markers with censoring rates above 53% were among those showing the most challenging correction profiles, supporting their value for identifying analytically compromised markers before batch correction.

Because *btma_quantnorm* was applied on the original scale in both analytical workflows, its output was identical across Strategies A and B, simplifying its implementation in this dataset. This characteristic reduces uncertainty regarding transformation choices and facilitates comparability across studies regardless of whether log_2_ preprocessing is applied. In contrast, *btma_quantreg* showed near-optimal ICC values for the Immune Checkpoint Panel in Strategy A but an extreme CV for the Essential Cytokines Panel (421.2%), consistent with back-transformation instability for near-LOD measurements — a pattern not captured by ICC alone and only identified through the inter-day CV metric. This finding highlights the importance of using multiple complementary metrics, as ICC reduction alone may obscure undesirable effects on the scale or variability of corrected concentrations.

*Btma_standardize* and *btma_ipw* represent distinct correction approaches when covariates are supplied: the former relies on standardization-based adjustment, whereas the latter uses inverse-probability weighting. However, in the analysis presented here, no additional covariates were supplied to either method, because all methods were compared under the same covariate-free benchmarking framework. Under this specification, both procedures reduced operationally to the same batch-only mean adjustment applied by *btma_simple*, resulting in identical corrected values. This was verified against the behavior of the *batchtma* package and reflects the analytical specification used here rather than an error in the data or in the implementation.

Beyond the analytical dimension, these results carry practical implications for translational biomarker studies. In CRC specifically, markers such as sPD-1, sPD-L1, and sTIM-3 are being actively evaluated as indicators of immune context and as candidate biomarkers for patient stratification (31–33). If the data used in these analyses have not been harmonized, batch-driven differences may be mistaken for biology while true biological signals may be obscured by technical noise. Addressing this before any downstream interpretation is therefore essential for reliable biomarker analysis. Although this study was performed using the LEGENDplex™ platform, the principles evaluated may be relevant to other multiplex immunoassay platforms, such as Luminex xMAP or MSD, where comparable issues related to batch-structure, matrix complexity, and censoring have been described (16, 17).

Another contribution of this study is the development of BatchCorrector: a freely accessible, R Shiny-based web application that implements the evaluated framework into a practical resource for researchers. The application enables users to upload their own datasets, apply and compare the evaluated correction methods and preprocessing strategies, visualize correction performance and export harmonized datasets for subsequent analysis. This application may facilitate the assessment and correction of batch effects in immunological studies with limited access to statistical expertise or local computational infrastructure. The underlying software is protected by registered intellectual property rights (registration number 2026RTE00927504). However, the application has not yet been externally validated beyond the dataset analyzed in this study. Its performance and reproducibility across independent datasets, as well as its usability in routine analytical practice, therefore, remain to be established. Consequently, the web application should be regarded as an accessible implementation of the evaluated framework, rather than as evidence that the selected method has been extensively validated across a range of analytical contexts. Prospective evaluation in independent cohorts, preferably within multicenter studies, will be necessary before the application can be recommended for widespread use.

Several strengths of this study merit consideration. To our knowledge, this is among the first systematic comparisons focused on clinical BBMI panels with substantial LOD-related censoring, using complementary performance metrics to avoid the potentially misleading conclusions associated with single-metric evaluations. Furthermore, the structured evaluation matrix (7 methods × 2 strategies × 2 panels × 3 metrics) provides an internally consistent evaluation of method behavior, while bootstrap-derived 95% confidence intervals for ICC estimates offer a transparent assessment of statistical uncertainty. In addition, the unequal batch sizes and censoring profile reflect challenges commonly encountered in real-world multi-batch immunoassay studies. The integration of these analyses into a web-based tool further increases the practical applicability of the proposed framework.

An important design limitation concerns the identifiability of technical and biological sources of between-batch variation. Because each analytical batch comprised a different, non-overlapping subset of patients and no common reference or replicate clinical samples were analyzed across batches, the variance attributed to batch membership cannot be interpreted as a pure estimate of analytical variability. Rather, it represents batch-associated variance that may include instrument-, reagent-, day- and pre-analytical effects together with residual biological heterogeneity between patient subsets. In this regard, although the aliquots used for the present analysis were thawed once immediately before measurement, storage duration differed across analytical batches (**Supplementary Table 1**), and analytical day, LEGENDplex™ reagent lot and operator were partially or completely aligned with batch membership. These factors therefore represent real-world pre-analytical and analytical sources of variability whose individual contributions could not be disentangled retrospectively from the overall batch-associated component. The absence of significant differences in the measured demographic and clinicopathological characteristics across batches reduces the likelihood of major clinical confounding but cannot exclude heterogeneity related to unmeasured variables, such as inflammatory status or pre-analytical conditions. This consideration is particularly relevant to distribution-matching procedures such as quantile normalization, which may attenuate genuine biological differences if these are confounded with batch allocation. Future studies should therefore include pooled reference material or replicate aliquots distributed across analytical runs to enable direct estimation of the technical component of variance. This identifiability limitation applies equally to every method evaluated, since no single metric could separate technical from biological variation for any of them. For this reason, correction performance was assessed against several criteria — the intraclass correlation coefficient, Kruskal-Wallis significance, coefficient of variation, consistency across preprocessing strategies, and the signal-preservation criteria described above — rather than any one indicator. The resulting comparison between methods is conditional on these metrics and does not, on its own, establish which method most accurately removes technical rather than biological variation; it reflects relative performance under the metrics evaluated here, not an absolute measure of technical-correction accuracy. Although this study has some limitations, it also provides a useful basis for future research. The number of analytical batches was limited to three, which may restrict the precision of variance component estimation. A simulation-based sensitivity analysis exploring two to five analytical batches, with varying degrees of batch balance, showed that the main comparative findings were broadly consistent across this range (**Supplementary Table 10**-**12**), which partially addresses, but does not eliminate, this limitation. In addition, the analyses were performed in a single clinical cohort from a single laboratory using a single multiplex platform. These factors limit the generalizability of the findings to other cohorts, laboratories, platforms and batch structures. External validation in independent datasets, laboratories, and alternative immunoassay formats is warranted. Finally, the performance and usability of the ‘BatchCorrector’ application should be evaluated in independent real-world datasets before broader implementation.

## 5 Conclusions

Among seven batch-correction methods evaluated in 127 clinical samples, comprising for 25 BBMI-derived biomarkers measured across three batches, *btma_quantnorm* achieved the most balanced and reproducible reduction in batch-related variability for both cytokine and soluble immune checkpoint panels, irrespective of the preprocessing strategy applied. Beyond aggregate batch-effect metrics, a dedicated evaluation of biological signal preservation, including clinical associations, within-batch sample ranking, and marker correlation structure, indicated that *btma_quantnorm* performed within the same range as the alternative correction methods evaluated, providing complementary evidence, together with the simulation-based sensitivity analysis, supporting its use for downstream analyses under the conditions evaluated here. Its non-parametric distribution-matching framework provides robustness in the presence of LOD censoring, a common feature of clinical BBMI datasets, while its independence from preprocessing may facilitate implementation in multi-batch immunoassay workflows.

These findings support *btma_quantnorm* as a practical harmonization approach for clinical BBMI datasets affected by inter-batch variability under the analytical conditions evaluated here, rather than as a universal default method. The evaluation framework presented here, which includes LOD censoring assessment, multiparametric performance assessment, and dual-preprocessing strategy comparison, provides a reusable blueprint for future comparisons of batch-correction strategies in this analytical context (21). In addition, the ‘BatchCorrecor’ web application is a freely accessible resource that allows users to apply, compare and export batch-correction strategies in their own datasets.

## Data Availability

The original contributions presented in the study are included in the article/**Supplementary Material**. Further inquiries can be directed to the corresponding author/s.
