# Supplementary material for: Benchmarking batch-effect correction in clinical bead-based multiplex immunoassays: a framework and web tool for cytokine and soluble immune checkpoint data
Source: Front Immunol. 2026 Oct 8;17:1944271. doi: 10.3389/fimmu.2026.1944271 (PMC13687280; doi:10.3389/fimmu.2026.1944271)
Supplement: Supplementary file 1 [file Table1.docx]

***Supplementary Material***

1 **Supplementary Figures**

**Supplementary Figure 1. Inter-day coefficient of variation before and after *btma_quantnorm* correction.** Heatmaps showing the coefficient of variation (CV%) for each marker across the three analytical batches before and after batch correction. (**A**) Original, uncorrected data from the Essential Immune Response Panel and from (**B**) the Immune Checkpoint Panel. (**C**) Essential Immune Response Panel and (**D**) Immune Checkpoint Panel after *btma_quantnorm* correction. Values inside each cell indicate the inter-day CV (%) for each marker and batch. Colour intensity represents CV magnitude, with darker shades indicating higher variability.

2 **Supplementary Tables**

**Supplementary Table 1. Clinicopathological characteristics and available pre-analytical characteristics of colorectal cancer patients enrolled in this study.** Statistical analysis was performed using one-way ANOVA for age and storage duration. Categorical variables were compared using χ² test or Fisher’s exact test, as appropriate. For contingency tables longer than 2 x 2 with low expected counts, the Fisher–Freeman–Halton exact test was used. N/A values were excluded from statistical comparisons.

|  | **Total** | **Batch 1** | **Batch 2** | **Batch 3** | | ***p* value** | | |
| --- | --- | --- | --- | --- | --- | --- | --- | --- |
| **Number of individuals, (%)** | 127 | 26 (20.47) | 72 (56.69) | 29 (22.83) | | - | | |
| **Age in years, median (min-max)** | 72 (33-91) | 75 (53-91) | 72 (33-91) | 70 (33-90) | | 0.335 | | |
| **Biological sex, *n* (%)** |  |  |  |  | |  | | |
| Male | 81 (63.78) | 17 (65.38) | 47 (65.28) | 17 (58.62) | | 0.805 | | |
| Female | 46 (36.22) | 9 (34.62) | 25 (34.72) | 12 (41.38) | |  |  |  |
| **Tumour site, *n* (%)** |  |  |  |  | |  | | |
| Cecum | 21 (16.54) | 6 (23.08) | 11 (15.28) | 4 (13.79) | | 0.722 | | |
| Ascending colon | 28 (22.05) | 6 (23.08) | 14 (19.44) | 8 (27.59) | |  |  |  |
| Transverse colon | 9 (7.09) | 1 (3.85) | 4 (5.56) | 4 (13.79) | |  |  |  |
| Descending colon | 9 (7.09) | 0 (0.00) | 7 (9.72) | 2 (6.90) | |  |  |  |
| Sigmoid colon | 45 (35.43) | 10 (38.46) | 26 (36.11) | 9 (31.03) | |  |  |  |
| Rectum | 15 (11.81) | 3 (11.54) | 10 (13.89) | 2 (6.90) | |  |  |  |
| **TNMª stage, *n* (%)** |  |  |  |  | |  | | |
| I | 25 (19.69) | 6 (23.08) | 14 (19.44) | 5 (17.24) | | 0.922 | | |
| II | 54 (42.52) | 12 (46.15) | 29 (40.28) | 13 (44.83) | |  |  |  |
| III | 40 (31.50) | 7 (26.92) | 25 (34.75) | 8 (27.59) | |  |  |  |
| IV | 8 (6.30) | 1 (3.85) | 4 (5.56) | 3 (10.34) | |  |  |  |
| **ASA score, *n* (%)** |  |  |  |  | |  | | |
| 1 | 5 (3.94) | 0 | 4 (5.56) | 1 (3.45) | | 0.171 | | |
| 2 | 59 (46.46) | 10 (38.46) | 32 (44.44) | 17 (58.62) | |  |  |  |
| 3 | 54 (42.52) | 16 (61.54) | 28 (38.89) | 10 (34.48) | |  |  |  |
| 4 | 9 (7.09) | 0 | 8 (11.11) | 1 (3.45) | |  |  |  |
| **Perineural invasion, *n* (%)** |  |  |  |  | |  | | |
| Yes | 38 (29.92) | 11 (42.31) | 20 (27.78) | 7 (24.14) | | 0.336 | | |
| No | 83 (65.35) | 15 (57.69) | 46 (63.89) | 22 (75.86) | |  |  |  |
| N/A | 6 (4.72) | 0 | 6 (8.33) | 0 | |  |  |  |
| **Lymphovascular invasion, *n* (%)** |  |  |  |  | |  | | |
| Yes | 54 (42.52) | 9 (34.62) | 29 (40.28) | 16 (55.17) | | 0.305 | | |
| No | 67 (52.76) | 17 (65.38) | 37 (51.39) | 13 (44.83) | |  |  |  |
| N/A | 6 (4.72) | 0 | 6 (8.33) | 0 | |  |  |  |
| **Comorbidities, *n* (%)** |  |  |  |  | |  | | |
| Type II Diabetes mellitus | 32 (25.20) | 7 (26.92) | 17 (23.61) | 8 (27.59) | | 0.894 | | |
| Dyslipidaemia | 50 (39.37) | 13 (50.00) | 25 (34.72) | 12 (41.38) | | 0.410 | | |
| Arterial hypertension | 63 (49.61) | 14 (53.85) | 32 (44.44) | 17 (58.62) | | 0.387 | | |
| **Metastasis, *n* (%)** |  |  |  |  | |  | | |
| Yes | 15 (11.81) | 4 (15.38) | 8 (11.11) | 3 (10.34) | | 0.808 | | |
| No | 112 (88.19) | 22 (84.62) | 64 (88.89) | 26 (89.66) | |  |  |  |
| **Storage duration,**  **median (min-max)** |  |  |  |  | |  | | |
|  | 261 (15-496) | 65 (15-101) | 374 (258-496) | | 155 (65-221) | | 0.0001 |  |

**Supplementary Table 2. Censoring diagnostics table.**

| **Marker** | **Repeated Value** | **% Samples** | **Number of zeros** | **Censoring Type** |
| --- | --- | --- | --- | --- |
| IL-4 (A4) | 2.200 | 44.9 | 16 | Limit of detection (LOD) |
| IL-2 (A5) | 0.400 | 28.3 | 24 | Limit of detection (LOD) |
| IL-1β (A7) | 6.160 | 36.2 | 14 | Limit of detection (LOD) |
| TNF-α (A8) | 4.543 | 52.8 | 17 | Limit of detection (LOD) |
| IL-17A (B2) | 0.180 | 29.9 | 22 | Limit of detection (LOD) |
| IL-10 (B4) | 0.500 | 18.9 | 13 | Limit of detection (LOD) |
| IFN-γ (B5) | 7.163 | 47.2 | 11 | Limit of detection (LOD) |
| IL-12p70 (B6) | 3.200 | 46.5 | 16 | Limit of detection (LOD) |
| TGF-β1 Free Active (B9) | 63.770 | 55.1 | 28 | Limit of detection (LOD) |
| TGF-β1 (pg/mL) | 54.580 | 55.1 | 8 | Possible censoring |
| CTLA-4 | 10.340 | 53.5 | 9 | Limit of detection (LOD) |
| PD-L2 (B4) | 5000 | 57.5 | 0 | Upper limit of quantification (ULOQ) |

**Supplementary Table 3.** **Description of the batch-effect correction methods compared in the study.**

| **Method** | **Underlying Principle** | **Statistical Approach** | **Key Assumptions** |
| --- | --- | --- | --- |
| **Median centring** | Each batch is shifted so that its median matches a common reference value — typically the overall median computed across all batches. It is probably the simplest approach available and makes no assumption about the shape of the distribution. | A scalar offset *δ*b is estimated for each batch as the difference between the batch median and the grand median, and subtracted from all observations in that batch: *x′ij = xij − median(xb)*. No adjustment is made to the spread of values. | Batch effects are purely additive and do not vary across the concentration range. Within-batch variability is assumed to be comparable between batches. |
| **btma_simple** (mean-centring) | Conceptually identical to median centering but replaces the median with the arithmetic mean as the location parameter. This makes it somewhat more sensitive to outliers or skewed distributions, which is worth bearing in mind in clinical laboratory data. | Each observation is corrected by subtracting the batch-specific mean: *x′ij = xij − x̄b*. This is equivalent to fitting an additive fixed-effects model with batch as a single categorical predictor. | Batch effects act additively and the mean provides a reliable summary of the batch location (i.e., no severe skewness or heavy-tailed distributions). |
| **btma_standardise** (within-batch *z*-score) | Marginal standardisation: batch-specific means are adjusted for any supplied confounders (or estimated from batch alone if none are supplied), so that between-batch differences not attributable to biology are removed | Adjusted batch means are obtained from a linear regression model with batch and any supplied confounders as predictors; biomarker values are re-centred using these confounder-adjusted marginal means in place of the raw batch means | Batch effects comprise both an additive shift and a multiplicative scaling component. Within-batch distributions are assumed to be approximately normal — or at least sufficiently symmetric for the standard deviation to be a meaningful dispersion measure. |
| **ComBat** | An empirical Bayes method originally developed for genomics data that estimates both additive and multiplicative batch parameters for each measured feature. By borrowing information across features, it stabilizes parameter estimates, which is particularly useful when individual batches contain few samples. | The model decomposes the observed value as: *Yijg = αg + Xβg + γig + δig εijg*, where *γig* and *δig* represent the additive and multiplicative batch effects for feature *g* in batch *i*. Prior distributions are estimated empirically from the data, and the batch parameters are then shrunken towards these priors to obtain final corrected values. | The association between biological covariates and the outcome is linear. Batch effects have both an additive and a multiplicative component. The empirical prior distributions estimated from the data provide a reasonable approximation of the true distribution of batch parameters across features. |
| **btma_ipw** (inverse probability weighting) | Inverse-probability weighting: rather than adjusting raw batch means directly, this method estimates marginal (confounder-adjusted) batch means using probability weights, and re-centres biomarker values accordingly | Batch propensity scores — P(batch = b \| confounders) — are estimated via multinomial logistic regression (or are uniform if no confounders are supplied); stabilised inverse-probability weights are then used to compute a weighted marginal mean per batch, and biomarker values are re-centred using these weighted means. | All covariates determining batch assignment must be measured and included in the propensity model (no unmeasured confounding). The positivity assumption must hold: every unit must have a non-negligible probability of belonging to each batch. |
| **btma_quantreg** (quantile regression) | Extends the idea of mean-centering by modelling batch effects at multiple quantiles of the distribution simultaneously. This allows corrections that are not uniform across the measurement range — for instance, a batch may inflate high values but not low ones. | For a set of quantiles *τ*, a separate regression model is fitted at each quantile: *Qτ(Y \| batch) = αb,τ*. Observations are then corrected by aligning quantile-specific intercepts to a reference distribution, with interpolation applied between estimated quantiles. | Batch effects are heterogeneous across the distribution (i.e., they cannot be adequately captured by a single additive constant). A sufficient number of observations per batch is required to reliably estimate effects at extreme quantiles. |
| **btma_quantnorm** (quantile normalisation) | The most stringent of the methods evaluated: it forces all batches to have identical empirical distributions, effectively removing all between-batch distributional differences regardless of their origin. Originally developed for microarray data normalisation. | Within each batch, observations are ranked. Each rank is then assigned the mean of that rank's values across all batches: *x′(r) = mean(x(r),1, …, x(r),B)*. Tied ranks receive the average of the corresponding mean values. | The true biological distribution of the analyte is identical across batches — any observed between-batch differences in distribution are assumed to be purely technical in origin. This is a strong assumption that may not hold when batch assignment is related to patient characteristics. |

**IPW**, inverse probability weighting; SD, standard deviation; *btma*, batch TMA (tissue microarray) correction methods.

**Supplementary Table 4. Descriptive statistics of the sample studied.**

| Marker | Batch 1 - Median (P25–P75) | | Batch 2 - Median (P25–P75) | Batch 3 - Median (P25–P75) | p-value |  |  |
| --- | --- | --- | --- | --- | --- | --- | --- |
| **Essential Immune Response Panel** | |  |  |  |  |  |  |
| IL-4 | 0.48 (0.48–0.80) | | 2.20 (2.20–2.20) | 0.00 (0.00–0.09) | **<0.001** |  |  |
| IL-2 | 0.34 (0.34–0.38) | | 0.43 (0.40–0.47) | 0.00 (0.00–0.00) | **<0.001** |  |  |
| CXCL10 | 42.74 (22.43–85.52) | | 177.17 (132.86–266.60) | 662.23 (503.11–802.65) | **<0.001** |  |  |
| IL-1β | 0.92 (0.92–1.08) | | 6.16 (6.16–10.33) | 4.75 (0.00–11.27) | **<0.001** |  |  |
| TNF-α | 0.54(0.54–0.58) | | 4.54 (4.54–4.54) | 0.00 (0.00–111.00) | **<0.001** |  |  |
| CCL2 | 20.22 (9.85–23.26) | | 59.85 (36.18–98.12) | 128.66 (50.92–263.52) | **<0.001** |  |  |
| IL-17A | 1.00 (1.00–1.05) | | 0.18 (0.18–0.60) | 0.00 (0.00–0.00) | **<0.001** |  |  |
| IL-6 | 0.78 (0.47–0.99) | | 3.73 (1.81–6.42) | 0.94 (0.12–3.78) | **<0.001** |  |  |
| IL-10 | 0.50 (0.50–0.50) | | 1.20 (0.75–1.95) | 0.05 (0.00–0.75) | **<0.001** |  |  |
| IFN-γ | 0.66(0.66–0.67) | | 7.16 (7.16–7.16) | 1.98 (0.00–4.23) | **<0.001** |  |  |
| IL-12p70 | 0.50 (0.50–0.64) | | 3.20 (3.20–3.20) | 0.00 (0.00–0.69) | **<0.001** |  |  |
| IL-8 | 0.64 (0.50–1.04) | | 5.74 (2.61–12.93) | 7.50 (2.04–33.64) | **<0.001** |  |  |
| Free Active TGF-β | 4.25 (4.25–4.25) | | 63.77 (63.77–63.77) | 0.00 (0.00–0.00) | **<0.001** |  |  |
| **Immune Checkpoint Panel** |  |  |  |  |  |  |  |
|  |  | | | | | | |
| sCD25 | 169.49 (106.94–403.95) | | 999.48 (743.54–1630.76) | 866.91 (608.07–1174.04) | **<0.001** |  |  |
| s4-1BB | 3.76 (1.97–5.72) | | 13.76 (9.51–23.50) | 30.73 (22.58–38.96) | **<0.001** |  |  |
| sCD27 | 15732.90 (8293.20–33150.85) | | 52501.25 (39346.88–116393.02) | 27952.70(19988.70–49636.50) | **<0.001** |  |  |
| sCD86 | 43.50 (27.55–53.10) | | 246.13 (183.85–317.85) | 170.77 (137.66–264.52) | **<0.001** |  |  |
| sTGF-β | 3.32 (3.32–3.32) | | 54.58 (54.58–54.58) | 5.64 (0.00–22.97) | **<0.001** |  |  |
| sCTLA4 | 0.36 (0.31–1.49) | | 10.34 (10.34–10.34) | 0.07 (0.00–1.34) | **<0.001** |  |  |
| sPD-L1 | 9.44 (4.68–16.44) | | 94.01 (76.30–150.33) | 207.41 (136.14–279.28) | **<0.001** |  |  |
| sPD-L2 | 4668.68(4200.07–5000.00) | | 5000.00 (5000.00–5000.00) | 4319.72 (3660.73–4613.45) | **<0.001** |  |  |
| sPD-1 | 1.99 (0.87–4.53) | | 12.27 (8.16–21.66) | 25.91 (17.79–29.94) | **<0.001** |  |  |
| sTIM-3 | 4623.62 (2385.30–16418.86) | | 26343.22 (14726.23–40341.21) | 10641.25 (5886.82–16654.20) | **<0.001** |  |  |
| sLAG3 | 373.45 (252.00–679.80) | | 1230.50 (860.50–1783.80) | 794.20 (615.60–960.50) | **<0.001** |  |  |
| Galectin-9 (B9) | 15152.30 (9599.55–24353.65) | | 55059.90 (44185.53–70336.70) | 26612.90 (17901.60–30003.30) | **<0.001** |  |  |

**Supplementary Table 5. Clinical effect-size preservation**

| **Method** | **Vector correlation (ρ)** | **Median \|Δρ\|** | **% same direction** | **Markers evaluated** |
| --- | --- | --- | --- | --- |
| ComBat | 0.837 | 0.0454 | 81.5% | 25/25 |
| btma_simple | 0.837 | 0.0453 | 82.5% | 25/25 |
| btma_standardize | 0.837 | 0.0453 | 82.5% | 25/25 |
| btma_ipw | 0.837 | 0.0453 | 82.5% | 25/25 |
| Median | 0.794 | 0.0508 | 79.3% | 25/25 |
| btma_quantnorm | 0.785 | 0.0514 | 76.7% | 25/25 |
| btma_quantreg | 0.863 | 0.0532 | 80.6% | 15/25* |

*Vector correlation: Pearson correlation between the 275 marker–clinical-variable Spearman ρ values (25 markers × 11 clinical variables) computed before vs. after correction; values closer to 1 indicate better preservation of clinical associations. *btma_quantreg failed to converge for 10 of 25 markers (IL-4, IL-2, TNF-α, IL-17A, IL-10, IFN-γ, IL-12p70, TGF-β, sTGF-β, sCTLA-4 — the markers most affected by censoring at the limit of detection); its statistics are computed on the remaining 15 markers only and are not directly comparable to the other six methods, which cover all 25 markers.*

**Supplementary Table 6. Within-batch rank preservation**

| **Method** | **Mean within-batch Spearman ρ** | **Markers evaluated** |
| --- | --- | --- |
| ComBat | 0.9997 | 25/25 |
| btma_simple | 0.9980 | 25/25 |
| btma_standardize | 0.9980 | 25/25 |
| btma_ipw | 0.9980 | 25/25 |
| Median | 0.9997 | 25/25 |
| btma_quantnorm | 0.9998 | 25/25 |
| btma_quantreg | 0.9999 | 15/25* |

*Spearman correlation between original and corrected values computed separately within each of the 3 analytical batches (days), then averaged per marker and across markers. All methods preserve within-batch sample ranking essentially perfectly (ρ ≥ 0.998).*

**Supplementary Table 7. Marker correlation-structure preservation**

| **Method** | **All 25 markers** | **Essential Cytokines (13)** | **Immune Checkpoint (12)** |
| --- | --- | --- | --- |
| ComBat | 0.439 | 0.601 | 0.547 |
| btma_simple | 0.437 | 0.716 | 0.547 |
| btma_standardize | 0.437 | 0.716 | 0.547 |
| btma_ipw | 0.437 | 0.716 | 0.547 |
| Median | 0.423 | 0.737 | 0.551 |
| btma_quantnorm | 0.368 | 0.725 | 0.488 |
| btma_quantreg | 0.323 | 0.428 | 0.485 |

*Matrix correlation coefficient: Pearson correlation between the upper-triangle entries of the marker-marker Spearman correlation matrices computed before vs. after correction (higher = better preserved correlation structure).*

**Supplementary Table 8.** **Summary of marker–clinical association preservation before and after *btma_quantnorm* correction.** Normality was assessed using the Shapiro-Wilk test; associations were evaluated using Spearman’s rank correlation coefficient; statistical significance was defined as *p*<0.05.

| **Clinical variable** | **Significant before** | **Significant after** | **Maintained** | **Attenuated/lost** | **New after correction** | **Non-significant both** |
| --- | --- | --- | --- | --- | --- | --- |
| *Exitus* | 0 | 2 | 0 | 0 | 2 | 23 |
| Age in years | 6 | 9 | 5 | 1 | 4 | 15 |
| Biological sex | 0 | 1 | 0 | 0 | 1 | 24 |
| Tumour site | 0 | 1 | 0 | 0 | 1 | 24 |
| TNMª stage | 1 | 3 | 1 | 0 | 2 | 22 |
| ASA score | 5 | 10 | 4 | 1 | 6 | 14 |
| Perineural invasion | 5 | 1 | 1 | 4 | 0 | 20 |
| Lymphovascular invasion | 1 | 2 | 0 | 1 | 2 | 22 |
| Type II Diabetes mellitus | 0 | 3 | 0 | 0 | 3 | 22 |
| Dyslipidaemia | 1 | 1 | 0 | 1 | 1 | 23 |
| Arterial hypertension | 0 | 5 | 0 | 0 | 5 | 20 |

'Maintained' indicates associations that were significant before and after correction; 'attenuated/lost' indicates associations that were significant before correction only; 'new after correction' indicates associations that were significant after correction only; and 'non-significant both' indicates associations that were not significant in either dataset.

**Supplementary Table 9. Marker-level clinical associations modified or preserved after *btma_quantnorm* correction.** Normality was assessed using the Shapiro-Wilk test; associations were evaluated using Spearman’s rank correlation coefficient; statistical significance was defined as *p*<0.05.

| **Clinical variable** | **Marker** | **Panel** | **Rho before** | ***p-value* before** | **Rho after** | ***p*-value after** | **Interpretation** |
| --- | --- | --- | --- | --- | --- | --- | --- |
| *Exitus* | IFN-γ | EIRP | -0,092 | 0,305 | -0,196 | 0,027 | New after correction |
| *Exitus* | sPD-L2 | ICP | 0,157 | 0,079 | 0,216 | 0,015 | New after correction |
| Age in years | CXCL10 | EIRP | 0,073 | 0,418 | 0,267 | 0,002 | New after correction |
| Age in years | IL-1β | EIRP | -0,177 | 0,047 | -0,086 | 0,334 | Attenuated/lost |
| Age in years | IL-6 | EIRP | 0,098 | 0,274 | 0,206 | 0,02 | New after correction |
| Age in years | IFN-γ | EIRP | -0,119 | 0,182 | -0,175 | 0,049 | New after correction |
| Age in years | TGF-β | EIRP | -0,022 | 0,807 | -0,178 | 0,046 | New after correction |
| Age in years | sCD25 | ICP | 0,243 | 0,006 | 0,387 | 0 | Maintained |
| Age in years | sCD27 | ICP | 0,305 | 0 | 0,37 | 0 | Maintained |
| Age in years | sTIM-3 | ICP | 0,335 | 0 | 0,416 | 0 | Maintained |
| Age in years | sLAG-3 | ICP | 0,24 | 0,007 | 0,352 | 0 | Maintained |
| Age in years | Galectin-9 | ICP | 0,187 | 0,035 | 0,329 | 0 | Maintained |
| Biological sex | sCD25 | ICP | -0,095 | 0,288 | -0,192 | 0,03 | New after correction |
| ASA score | CXCL10 | EIRP | 0,065 | 0,469 | 0,218 | 0,014 | New after correction |
| ASA score | IL-1β | EIRP | -0,161 | 0,07 | -0,266 | 0,002 | New after correction |
| ASA score | TNF-α | EIRP | -0,181 | 0,042 | -0,242 | 0,006 | Maintained |
| ASA score | IL-10 | EIRP | 0,216 | 0,015 | 0,173 | 0,052 | Attenuated/lost |
| ASA score | IFN-γ | EIRP | -0,018 | 0,841 | -0,179 | 0,045 | New after correction |
| ASA score | sCD25 | ICP | 0,118 | 0,186 | 0,237 | 0,007 | New after correction |
| ASA score | sCD27 | ICP | 0,247 | 0,005 | 0,33 | 0 | Maintained |
| ASA score | sPD-L2 | ICP | 0,221 | 0,012 | 0,203 | 0,022 | Maintained |
| ASA score | sTIM-3 | ICP | 0,32 | 0 | 0,383 | 0 | Maintained |
| ASA score | sLAG-3 | ICP | 0,162 | 0,069 | 0,233 | 0,008 | New after correction |
| ASA score | Galectin-9 | ICP | 0,109 | 0,224 | 0,186 | 0,037 | New after correction |
| Tumour site | IL-6 | EIRP | -0.058 | 0.517 | -0.195 | 0.028 | New after correction |
| TNMª stage | IL-8 | EIRP | -0.228 | 0.010 | -0.285 | 0.001 | Maintained |
| TNMª stage | IL-1β | EIRP | -0.151 | 0.091 | -0.189 | 0.034 | New after correction |
| TNMª stage | IL-12p70 | EIRP | -0.066 | 0.461 | -0.228 | 0.010 | New after correction |
| Perineural invasion | IL-8 | EIRP | -0,206 | 0,023 | -0,139 | 0,128 | Attenuated/lost |
| Perineural invasion | sCD86 | ICP | -0,212 | 0,019 | -0,16 | 0,08 | Attenuated/lost |
| Perineural invasion | sPD-L1 | ICP | -0,228 | 0,012 | -0,178 | 0,05 | Attenuated/lost |
| Perineural invasion | sPD-1 | ICP | -0,274 | 0,002 | -0,234 | 0,01 | Maintained |
| Perineural invasion | sLAG-3 | ICP | -0,192 | 0,035 | -0,172 | 0,059 | Attenuated/lost |
| Lymphovascular invasion | IL-17A | EIRP | -0,191 | 0,036 | -0,095 | 0,302 | Attenuated/lost |
| Lymphovascular invasion | IL-8 | EIRP | -0,16 | 0,079 | -0,211 | 0,02 | New after correction |
| Lymphovascular invasion | sPD-1 | ICP | -0,021 | 0,821 | -0,184 | 0,043 | New after correction |
| Lymphovascular invasion | IL-6 | EIRP | -0,058 | 0,517 | -0,195 | 0,028 | New after correction |
| Type II Diabetes mellitus | CCL2 | EIRP | -0,155 | 0,083 | -0,199 | 0,025 | New after correction |
| Type II Diabetes mellitus | IL-17A | EIRP | -0,159 | 0,075 | -0,187 | 0,035 | New after correction |
| Type II Diabetes mellitus | sTGF-β | ICP | 0,013 | 0,884 | 0,2 | 0,024 | New after correction |
| Dyslipidaemia | TNF-α | EIRP | -0,217 | 0,014 | -0,077 | 0,39 | Attenuated/lost |
| Dyslipidaemia | sPD-L1 | ICP | -0,173 | 0,051 | -0,175 | 0,049 | New after correction |
| Arterial hypertension | s4-1BB | ICP | 0,153 | 0,086 | 0,218 | 0,014 | New after correction |
| Arterial hypertension | sCD27 | ICP | 0,168 | 0,059 | 0,27 | 0,002 | New after correction |
| Arterial hypertension | sTIM-3 | ICP | 0,157 | 0,077 | 0,28 | 0,001 | New after correction |
| Arterial hypertension | sLAG-3 | ICP | 0,134 | 0,132 | 0,239 | 0,007 | New after correction |
| Arterial hypertension | Galectin-9 | ICP | 0,084 | 0,349 | 0,232 | 0,009 | New after correction |

'Maintained' indicates associations that were significant before and after correction; 'attenuated/lost' indicates associations that were significant before correction only; 'new after correction' indicates associations that were significant after correction only; and 'non-significant both' indicates associations that were not significant in either dataset. ICP, Immune Checkpoint Panel; EIRP, Essential Immune Response Panel.

**Supplementary Table 10. Description of the simulation scenarios used in the sensitivity analysis.** The table describes the eight simulation scenarios used to test the robustness of the correction-method comparison beyond the real dataset. Supplementary Table 11 reports correction performance for the six scenarios that vary censoring, batch structure and sample size (S1–S4, S6, S8), averaged across the two preprocessing pipelines and both marker panels. Supplementary Table 12 reports the two covariate scenarios (S10–S11), which instead quantify how much true biological signal is preserved when a covariate is either confounded with batch or independent of it.

| **Scenario** | **Design varied** | **Parameters** | **Purpose** |
| --- | --- | --- | --- |
| S1 | Censoring proportion | ~10% left-censored values per marker; 3 balanced batches (n=26/72/29); N=127. | Lower bound of the censoring range tested. |
| S2 | Censoring proportion | ~30% left-censored values per marker (matches the real cohort); 3 balanced batches (n=26/72/29); N=127. | Baseline scenario, replicating the censoring level of the real dataset. |
| S3 | Censoring proportion | ~50% left-censored values per marker; 3 balanced batches (n=26/72/29); N=127. | Upper bound of the censoring range tested. |
| S4 | Number of batches | ~30% censoring; 5 balanced batches (n=24/26/25/27/25); N=127 (unchanged). | Tests robustness when more analytical batches are used than in the real study. |
| S6 | Combined stress test | ~55% censoring; 5 unbalanced batches (n=10/45/20/40/12); stronger simulated batch effect. | Worst-case scenario, combining high censoring, batch imbalance and a stronger batch effect. |
| S8 | Sample size | ~30% censoring; 3 batches (n=82/227/91); N≈400 (~3× the real cohort). | Tests whether a larger sample changes the relative performance of the methods. |
| S10 | Covariate-batch confounding | Binary covariate with a true effect (+1 SD, log2 scale) on 3 markers; probability of the covariate given batch = 0.85/0.45/0.10. | Places an empirical bound on how much true biological signal is lost when a confounder is strongly tied to batch. |
| S11 | Covariate independent of batch | Same covariate and true effect on the same 3 markers; probability of the covariate given batch = 0.45/0.45/0.45 (equal across batches). | Control scenario with no batch-covariate confounding, to check that biological signal is preserved when the two are unrelated. |

**Supplementary Table 11. Batch-effect correction performance across simulation scenarios S1–S4, S6 and S8.**

| **Scenario** | **Method** | **Mean ICC (range)** | **Mean KW- significant markers** | **Mean CV (%)** | **Mean ICC reduction (%)** |
| --- | --- | --- | --- | --- | --- |
| **S1 — low censoring (~10%)** | **Original (uncorrected)** | 0.172 (0.086–0.330) | 10.0 | 135.3 | 0.0 |
|  | **Median** | 0.015 (0.001–0.142) | 0.0 | 388.6 | 91.3 |
|  | **ComBat** | 0.000 (0.000–0.091) | 0.5 | 116.2 | 100.0 |
|  | **btma_simple** | 0.000 (0.000–0.100) | 3.5 | 119.4 | 100.0 |
|  | **btma_standardize** | 0.000 (0.000–0.099) | 3.5 | 119.4 | 100.0 |
|  | **btma_ipw** | 0.000 (0.000–0.101) | 3.5 | 119.4 | 100.0 |
|  | **btma_quantreg** | 0.172 (0.085–0.329) | 10.0 | 135.3 | 0.0 |
|  | **btma_quantnorm** | 0.000 (0.000–0.080) | 0.0 | 111.5 | 100.0 |
| **S2 — baseline (~30%)** | **Original (uncorrected)** | 0.146 (0.069–0.305) | 8.0 | 136.0 | 0.0 |
|  | **Median** | 0.029 (0.005–0.164) | 1.0 | 473.3 | 81.7 |
|  | **ComBat** | 0.000 (0.000–0.094) | 1.3 | 116.0 | 100.0 |
|  | **btma_simple** | 0.001 (0.000–0.106) | 3.0 | 121.9 | 99.6 |
|  | **btma_standardize** | 0.001 (0.000–0.106) | 3.0 | 121.9 | 99.6 |
|  | **btma_ipw** | 0.001 (0.000–0.105) | 3.0 | 121.9 | 99.6 |
|  | **btma_quantreg** | 0.146 (0.070–0.303) | 8.0 | 136.0 | 0.0 |
|  | **btma_quantnorm** | 0.001 (0.000–0.087) | 0.0 | 110.2 | 99.7 |
| **S3 — high censoring (~50%)** | **Original (uncorrected)** | 0.080 (0.035–0.229) | 7.5 | 147.1 | 0.0 |
|  | **Median** | 0.028 (0.002–0.156) | 1.5 | 397.4 | 62.2 |
|  | **ComBat** | 0.000 (0.000–0.088) | 3.5 | 120.4 | 100.0 |
|  | **btma_simple** | 0.003 (0.000–0.120) | 6.3 | 134.7 | 96.1 |
|  | **btma_standardize** | 0.003 (0.000–0.120) | 6.3 | 134.7 | 96.1 |
|  | **btma_ipw** | 0.003 (0.000–0.122) | 6.3 | 134.7 | 96.1 |
|  | **btma_quantreg** | 0.080 (0.035–0.228) | 7.5 | 147.1 | 0.0 |
|  | **btma_quantnorm** | 0.000 (0.000–0.089) | 0.0 | 115.7 | 100.0 |
| **S4 — 5 batches** | **Original (uncorrected)** | 0.117 (0.063–0.280) | 10.0 | 128.1 | 0.0 |
|  | **Median** | 0.013 (0.005–0.142) | 0.5 | 350.2 | 88.5 |
|  | **ComBat** | 0.000 (0.000–0.091) | 0.8 | 112.4 | 100.0 |
|  | **btma_simple** | 0.000 (0.000–0.097) | 3.3 | 121.0 | 100.0 |
|  | **btma_standardize** | 0.000 (0.000–0.098) | 3.3 | 121.0 | 100.0 |
|  | **btma_ipw** | 0.000 (0.000–0.098) | 3.3 | 121.0 | 100.0 |
|  | **btma_quantreg** | 0.117 (0.063–0.279) | 10.0 | 128.1 | 0.0 |
|  | **btma_quantnorm** | 0.000 (0.000–0.070) | 0.0 | 102.0 | 100.0 |
| **S6 — worst case** | **Original (uncorrected)** | 0.168 (0.087–0.380) | 12.0 | 139.3 | 0.0 |
|  | **Median** | 0.043 (0.009–0.224) | 0.5 | 374.8 | 75.0 |
|  | **ComBat** | 0.040 (0.024–0.172) | 5.5 | 108.6 | 73.8 |
|  | **btma_simple** | 0.005 (0.000–0.162) | 5.3 | 115.4 | 97.1 |
|  | **btma_standardize** | 0.005 (0.000–0.161) | 5.3 | 115.4 | 97.1 |
|  | **btma_ipw** | 0.005 (0.000–0.162) | 5.3 | 115.4 | 97.1 |
|  | **btma_quantreg** | 0.168 (0.086–0.377) | 12.0 | 139.3 | 0.0 |
|  | **btma_quantnorm** | 0.000 (0.000–0.163) | 0.0 | 94.8 | 100.0 |
| **S8 — large sample (~3×)** | **Original (uncorrected)** | 0.157 (0.112–0.237) | 12.0 | 188.6 | 0.0 |
|  | **Median** | 0.034 (0.017–0.089) | 6.0 | 865.0 | 79.5 |
|  | **ComBat** | 0.000 (0.000–0.030) | 2.3 | 141.6 | 99.8 |
|  | **btma_simple** | 0.004 (0.001–0.055) | 4.8 | 151.2 | 97.5 |
|  | **btma_standardize** | 0.004 (0.001–0.054) | 4.8 | 151.2 | 97.5 |
|  | **btma_ipw** | 0.004 (0.001–0.054) | 4.8 | 151.2 | 97.5 |
|  | **btma_quantreg** | 0.157 (0.112–0.236) | 12.0 | 188.6 | 0.0 |
|  | **btma_quantnorm** | 0.000 (0.000–0.028) | 0.0 | 137.5 | 100.0 |

ICC, intraclass correlation coefficient (batch-associated); values are means across the two preprocessing pipelines (log2-transformed and original scale) and both marker panels (Essential Cytokines and Immune Checkpoints), with the range across these four combinations shown in parentheses. KW, number of markers per panel with a nominally significant Kruskal-Wallis test (p<0.05); ICC reduction, percentage reduction in mean ICC relative to the uncorrected (Original) data within the same scenario.

**Supplementary Table 12. Effect of covariate-batch confounding on batch-effect correction (simulation scenarios S10–S11).**

| **Scenario** | **Method** | **Mean ICC (batch)** | **Mean Cohen's d** | **Markers with significant association (n/3)** |
| --- | --- | --- | --- | --- |
| **S10 — confounded** | **Original (uncorrected)** | 0.1644 | 0.820 | 3 |
|  | **Median** | 0.0016 | 0.503 | 3 |
|  | **ComBat (no covariate)** | 0.0001 | 0.671 | 3 |
|  | **ComBat (with covariate)** | 0.0735 | 0.922 | 3 |
|  | **btma_simple** | 0.0001 | 0.645 | 3 |
|  | **btma_standardize** | 0.0001 | 0.636 | 3 |
|  | **btma_ipw** | 0.0001 | 0.645 | 3 |
|  | **btma_quantnorm** | 0.0001 | 0.685 | 3 |
| **S11 — orthogonal (control)** | **Original (uncorrected)** | 0.1613 | 0.849 | 3 |
|  | **Median** | 0.0291 | 0.571 | 3 |
|  | **ComBat (no covariate)** | 0.0001 | 0.888 | 3 |
|  | **ComBat (with covariate)** | 0.0001 | 0.891 | 3 |
|  | **btma_simple** | 0.0001 | 0.876 | 3 |
|  | **btma_standardize** | 0.0001 | 0.882 | 3 |
|  | **btma_ipw** | 0.0001 | 0.876 | 3 |
|  | **btma_quantnorm** | 0.0001 | 0.887 | 3 |

Mean ICC (batch), residual batch-associated intraclass correlation coefficient after correction. Mean Cohen's d, average effect size of the covariate on the 3 markers with a true simulated effect, after correction (higher values indicate more of the true biological signal was preserved). Markers with significant association, number of these 3 markers (out of 3) retaining a statistically significant covariate association after correction. ComBat is shown both without and with the covariate supplied as a model covariate; all batchtma-based methods (simple, standardize, ipw, quantnorm) were run without supplying the covariate, consistent with the rest of the analysis.
